# Visualizing vasculature and its response to therapy in the tumor microenvironment

**DOI:** 10.7150/thno.84947

**Published:** 2023-09-25

**Authors:** Qiaoya Lin, Peter L. Choyke, Noriko Sato

**Affiliations:** Molecular Imaging Branch, Center for Cancer Research, National Cancer Institute, National Institutes of Health, Bethesda, Maryland.

**Keywords:** intravital imaging, tumor vasculature, optical imaging, animal window models, vascular responses

## Abstract

Tumor vasculature plays a critical role in the progression and metastasis of tumors, antitumor immunity, drug delivery, and resistance to therapies. The morphological and functional changes of tumor vasculature in response to therapy take place in a spatiotemporal-dependent manner, which can be predictive of treatment outcomes. Dynamic monitoring of intratumor vasculature contributes to an improved understanding of the mechanisms of action of specific therapies or reasons for treatment failure, leading to therapy optimization. There is a rich history of methods used to image the vasculature. This review describes recent advances in imaging technologies to visualize the tumor vasculature, with a focus on enhanced intravital imaging techniques and tumor window models. We summarize new insights on spatial-temporal vascular responses to various therapies, including changes in vascular perfusion and permeability and immune-vascular crosstalk, obtained from intravital imaging. Finally, we briefly discuss the clinical applications of intravital imaging techniques.

## Introduction

**Figure 1:**
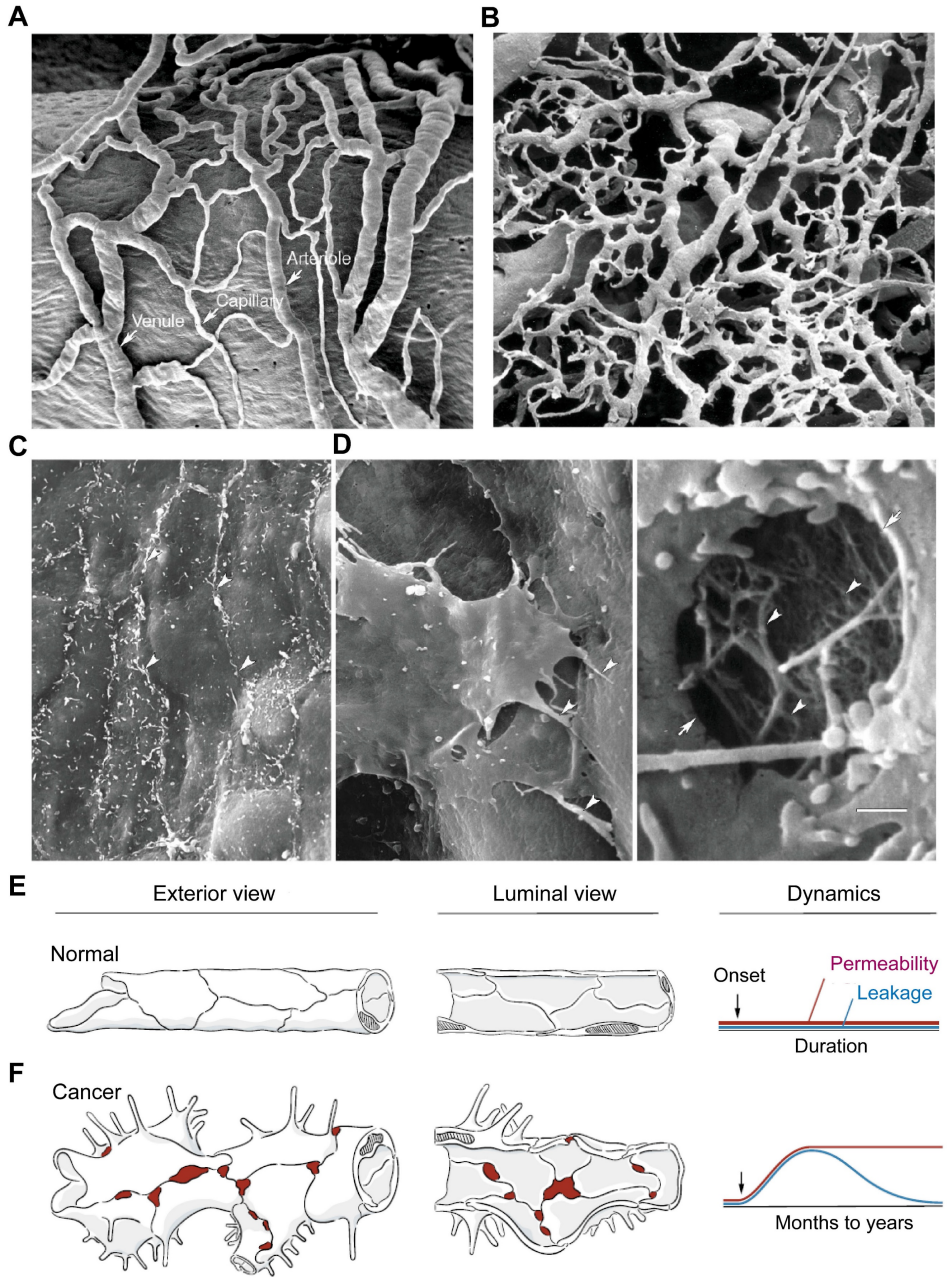
** Tumor vasculature are morphologically, structurally, and functionally abnormal.** (**A**) Scanning electron microscopic (SEM) imaging of normal microvasculature (vasa vasorum of rat carotid sinus) polymer cast shows an organized arrangement of arterioles, capillaries, and venules. (**B**) SEM image of a tumor microvasculature cast demonstrates disorganized vessels without the conventional hierarchy (i.e., arterioles, capillaries, and venules). A and B are adapted from 5, 8 with permission, copyright 2003 Springer Nature and Dr. Lametschwandtner. (**C**) SEM image of the luminal surface of normal blood vessel, which is smooth and has tight endothelial junctions (arrowheads, mouse mammary gland). (**D**) SEM of a tumor vessel shows widened intercellular spaces, overlapping endothelial cells, multiple cellular processes, and other abnormalities (left, arrowheads, MCaIV mouse mammary carcinoma). High magnification of a hole in the endothelium (arrows) shows the underlying basement membrane filaments (right, arrowheads). Scale bar in **D** applies to panels **A-D** (50 μm in **A** and **B**; 5 μm in **C**; 2 μm in **D** left; 0.5 μm in **D** right). Adapted from 5, 8 with permission, copyright 2003 Springer Nature. (**E, F**) Comparison of exterior and luminal views of blood vessels of the microcirculation and corresponding dynamics of permeability and leakage over time under normal conditions (**E**) and in cancer (**F**). In normal blood vessels, the permeability and leakage are low and stable. In cancer, increased endothelial permeability is sustained, but leakage decreases as interstitial pressure rises because of impaired lymphatic drainage. Modified from 7 with permission, copyright 2021 Elsevier.

Tumor vasculature differs from normal vasculature in morphology, structure, and function, and plays a critical role in tumor progression and metastasis 1, drug delivery 2, resistance to therapies 3, and antitumor immunity 4. In contrast to the normal blood vessels (Figure 1A), the tumor vasculature is strikingly heterogeneous and highly disorganized, lacking the hierarchy of vessel diameter (e.g. arterioles, capillaries, and venules) observed in normal tissues, as depicted in Figure 1B 5. In normal tissues, the endothelial barrier consists of two types of intercellular junctions: adherens junctions and tight junctions (Figure 1C). However, in tumor vessels, there are defective endothelial junctions with larger gaps between endothelial cells (Figure 1D), abnormal pericytes 6, and a lack of smooth muscles or basal membrane. The impaired endothelial barrier structure 7 creates a tumor microenvironment (TME) characterized by leaky vasculature and impaired blood flow, which result in hypoxia, low pH, and high interstitial fluid pressure.

**Figure 2:**
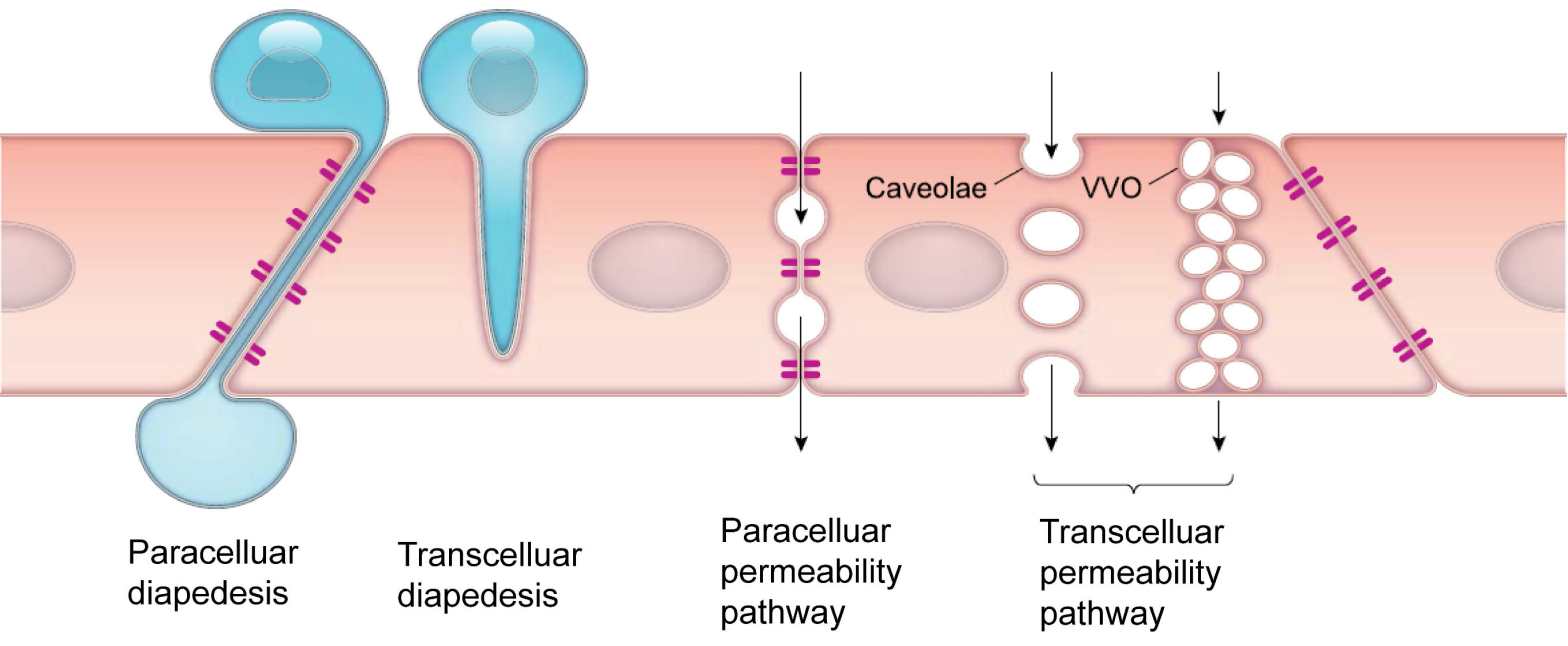
**Transendothelial pathways for cells and solutes.** Cells such as leukocytes can pass through the endothelial cell layer by paracellular and transcellular diapedesis. Endothelial permeability for macromolecules can be increased by opening endothelial junctions or through activation of vesicle-based transcellular pathways. VVO: vesiculo-vacuolar organelle. Modified from 9 with permission, copyright 2019 The American Physiological Society.

The endothelial barrier in the TME is a dynamic structure that controls the exchange of fluids and solutes, as well as various cells. Extravasation of cells and drugs occur via one of two types of mechanisms: paracellular and transcellular pathways (Figure 2) 9. Paracellular pathways are generated by gap junctions, where cells are initially slowed during transit but then migrate through the gap junction. The enhanced permeability and retention (EPR) effect, commonly associated with tumors has been a central paradigm for drug delivery since the late 1980s 10, however, the magnitude of the EPR effect in human tumors is often very small. EPR is related to inherent vascular leakage, poor lymphatic drainage, and slower blood flow in tumors, all of which favor the passage of macromolecules through paracellular inter-endothelial gaps, enhancing their passive diffusion and accumulation in the tumor. Recently, the contribution of the EPR effect to drug extravasation has been challenged by the discovery of transcellular extravasation as the primary route of transport for certain nanoparticles, such as gold nanoparticles (Figure 2) 11. Transcellular extravasation is mediated by endothelial vesicles that actively transports solutes across the endothelial cell, even against a gradient.

Abnormal tumor vasculature can also impede the transport and/or distribution of drugs, oxygen, and the infiltration of immune cells, such as T cells. As a result, abnormal tumor vasculature can contribute to resistance of tumor cells to chemotherapy, radiation therapy, and immunotherapy 12. Additionally, tumor endothelial cells can release several hypoxia-related factors such as vascular endothelial growth factor A (VEGF-A), which induces the expression of programmed cell death protein 1 (PD-1), T-cell immunoglobulin mucin-3 (TIM-3), and cytotoxic T-lymphocyte-associated protein 4 (CTLA-4) on CD8 T cells, rendering them dysfunctional. Alongside prostaglandin E2 (PGE2) and interleukin (IL)-10, VEGF-A induces Fas ligand (FasL) expression on endothelial cells, that affects the survival of effector T cells (rather than regulatory T cells (Tregs)). The expression of Clever-1/stabilin-1 on tumor vascular endothelial cells and hypoxia-related chemokine CCL-28 further aid the preferential recruitment of Tregs. Moreover, the hypoxic TME recruits monocytes that give rise to myeloid-derived suppressor cells (MDSCs) and tumor-associated macrophages (TAMs) and induces a differentially and functionally immature phenotype of DCs, collectively supporting an immunosuppressive TME. The more glycolytic metabolism of tumors results in acidification of the TME, thereby inhibiting cytotoxicity activities of CD8 T cells 4, 13-16.

Due to the critical roles of tumor vasculature in defining the TME, monitoring morphological and functional changes of the vasculature in response to therapies holds significant importance. Imaging techniques of vessels need to match both spatial and temporal scales to dynamically assess the tumor vasculature. As summarized in Table 1, in order to obtain sufficient spatial information, the method must be flexible enough to capture both the full extent of vessel sprouting (hundreds of millimeter resolution) as well as identify intercellular openings in tumors vasculatures (~70 nanometer resolution). Higher scanning speeds or temporal resolutions provide greater information for imaging changes in dynamic permeability. Functional imaging allows for assessment of perfusion, immune-vascular crosstalk (including immunocytes, cytokines, and vessel interactions) and oxygen saturation, all of which are equally important as structural analysis 7, 8, 17. Vascular imaging techniques should also possess the ability to record various functional events *in vivo* at multiscales 9. In this review, we describe recent advances in intravital tumor vascular imaging, covering imaging of vessels within tumors in live animals and humans. Focus will be put on *in vivo* real-time optical imaging technologies utilizing intravital microscopy (IVM) and animal window models. Clinical applications of these technologies are also discussed.

## Methods to Visualize Vasculature *In Vivo*

### Brief history of *in vivo* tumor vascular imaging

**Figure 3:**
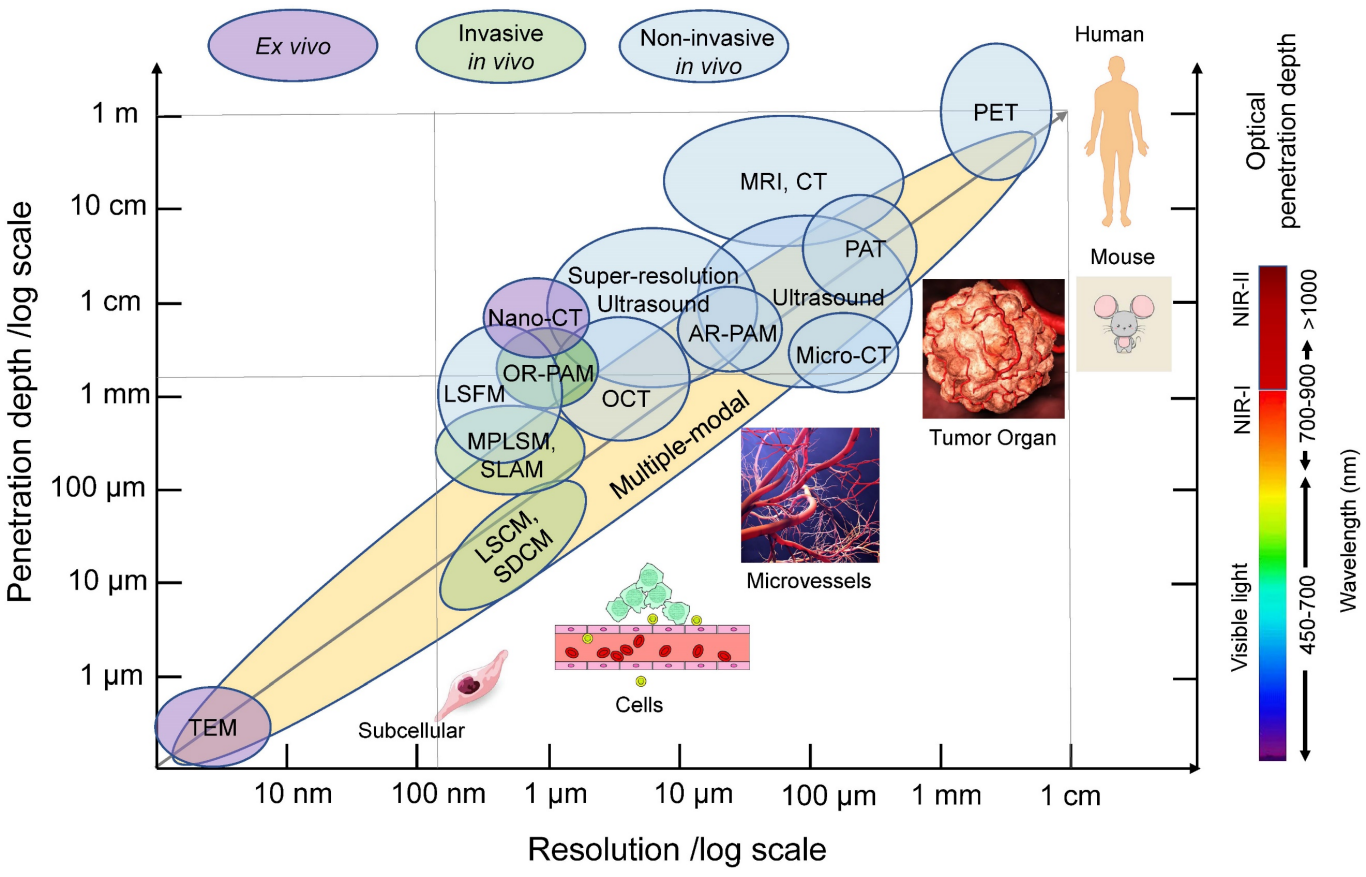
** Common *in vivo* tumor vascular imaging techniques and their resolution and penetration depth.** Non-invasive (blue circles) and invasive (green circles) *in vivo* imaging techniques and *ex vivo* imaging techniques (purple circles) for vascular imaging are shown in relation to their resolution and tissue penetration depth. Multi-modality imaging (yellow) takes advantage of combining techniques. The optical penetration depth increases as the wavelength increases from visible light, NIR-I to NIR-II. PAT: photoacoustic tomography, AR-PAM: acoustic-resolution photoacoustic microscopy, OCT: optical coherence tomography, LSFM: light-sheet fluorescence microscopy, OR-PAM: optical-resolution photoacoustic microscopy, MPLSM: multiphoton laser scanning microscopy, SLAM: simultaneous label-free autofluorescence-multiharmonic microscopy, LSCM: laser scanning confocal microscopy, SDCM: spinning disk confocal microscopy, TEM: Transmission electron microscopy, NIR: near-infrared.

In the past several decades, various imaging techniques, including computed tomography (CT), positron emission tomography (PET), magnetic resonance imaging (MRI), and ultrasound, have been developed and utilized to visualize tumor vasculature at different scales, both in preclinical and clinical settings. Previous reviews have covered the use of these modalities for imaging angiogenesis at different scales 5. With the evolution of those technologies, the spatial resolution of blood vessel imaging has dramatically increased. For instance, CT resolution has progressed from approximately 500 µm using CT angiography to 100 µm for microcomputed tomography (micro-CT), 50 µm for volumetric computed tomography 22, and even down to 400 nm for nano-computed tomography (nano-CT) (Figure 3). Current clinical and small animal PET scanners have a spatial resolution of approximately 1.5 mm-5 mm (Figure 3). MRI has the advantage of using magnetic fields rather than potentially harmful ionizing radiation for visualization. The spatial resolution of MRI has improved from hundreds of micrometers to 10 micrometers with micro-MRI 5 (Figure 3). Ultrasound, which does not involve ionizing radiation, is also commonly used to visualize vasculature. The resolution of conventional clinical ultrasound imaging is 200 µm~1 mm due to fundamental diffraction limits (Figure 3). Super-high resolution ultrasound with ultrafast frame rates (more than 500 frames per second), achieving resolutions of around 1 µm, has been developed 23, 24.

### Advances in intravital tumor vascular imaging

**Figure 4:**
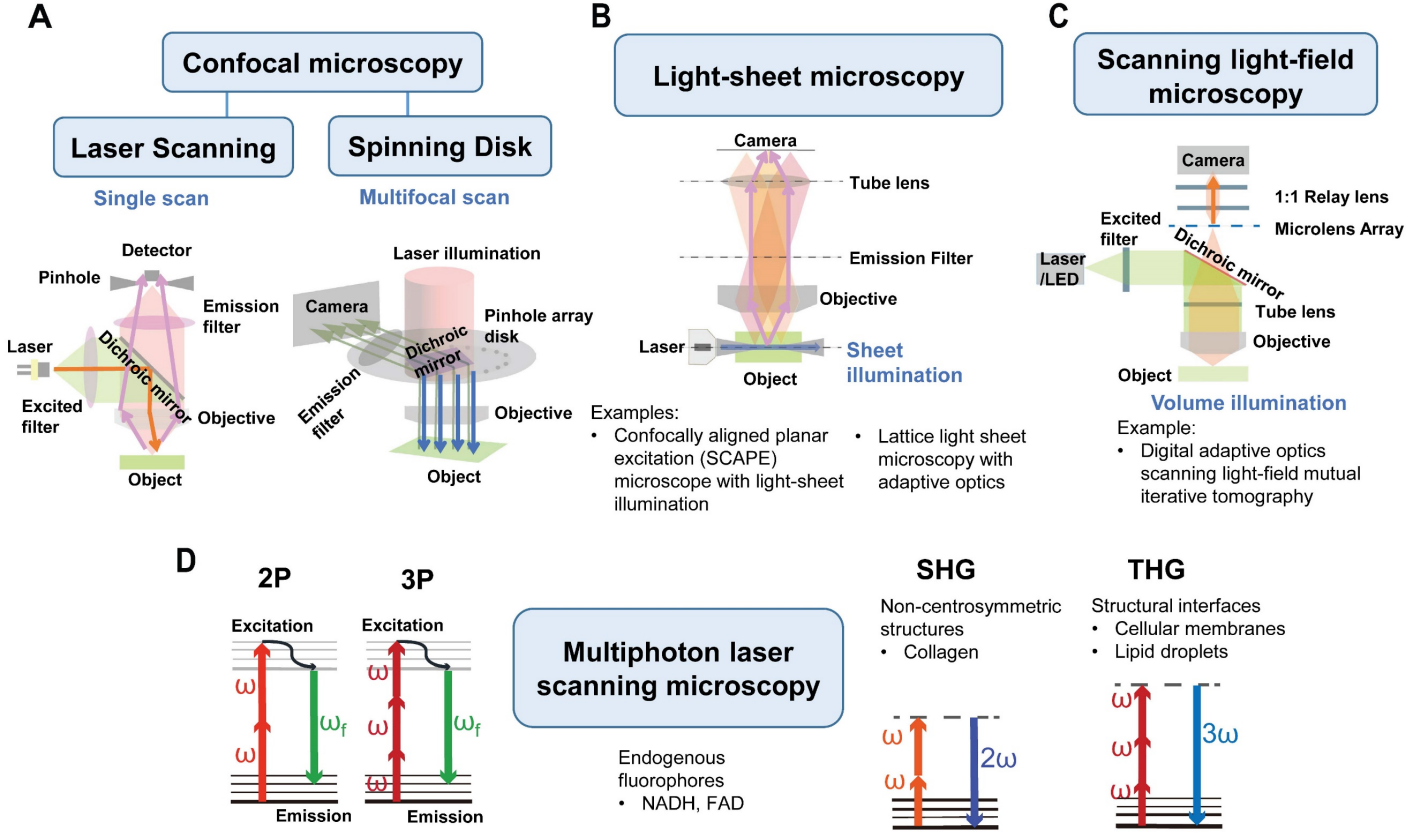
** IVM methods in tumor vascular imaging.** Current microscopies used in intravital tumor vascular imaging: (**A**) Confocal microscopy: laser scanning confocal microscopy (single scan type) and spinning disk confocal microscopy (multifocal scan type), (**B**) Light-sheet microscopy (sheet illumination or plane illumination), (**C**) Scanning light-field microscopy (volume illumination) and (**D**) Multiphoton laser scanning microscopy (MPLSM). (**A-C**) Schematic diagram of those microscopies. In (**A-C**), a fluorophore is excited by absorbing one single photon of a certain wavelength. (**D**) In MPLSM, the fluorophore is excited by simultaneously absorbing two photons (2P) or three photons (3P) of a longer wavelength. MPLSM can provide metabolic information by imaging endogenous fluorophores of nicotinamide dinucleotide (NADH) and flavin adenine dinucleotide (FAD). MPLSM allows label-free imaging by collecting second-harmonic generation (SHG) and third-harmonic generation (THG) signals. The SHG occurs at highly ordered non-centrosymmetric structures, such as fibrillar collagen, whereas the THG occurs at structural interfaces, such as cellular membranes and lipid droplets.

In recent years, development and improvements in optical imaging methods have facilitated improved imaging of the tumor vasculature *in vivo.* These methods offer higher resolution, deeper imaging depth, and greater metabolic information. With these techniques, it is now possible to image the heterogeneous tumor blood vessels at the single-cell level, even without the need for labeling. Additionally, dynamic changes within the vasculature can be recorded, providing valuable information on cell-to-cell interactions using multi-color imaging. This section focuses on highlighting the most recent advances in intravital tumor vascular imaging techniques, primarily intravital microscopy (IVM) methods, as depicted in Figure 4. Additionally, new animal window models will be discussed, which have contributed to these advancements.

#### IVM: Confocal microscopy and multiphoton laser scanning microscopy

The most commonly used methods in intravital imaging are confocal microscopy, including laser scanning confocal microscopy (LSCM) and spinning disk confocal microscopy (SDCM), as well as multiphoton laser scanning microscopy (MPLSM). Compared to wide-field fluorescence microscopy, LSCM addresses some of the scattering effects of fluorescent light by incorporating a pinhole aperture at the confocal plane in front of the detector, which rejects fluorescence from other planes (Figure 4A, left panel) 25. The spatial resolution can reach ~200 nm in the lateral dimension and ~500 nm in the axial dimension at ~30 frame per second (fps) scanning speed (Figure 3). This high-resolution enables visualization of fine tumor vessels *in vivo*. However, this imaging technique has a limited imaging depth (<~100 µm) and relatively high photobleaching and phototoxicity, leading to time limited imaging and/or tissue damage.

MPLSM utilizes the simultaneous nonlinear absorption of two-photons or three-photons generated by femtosecond pulsed lasers, to excite fluorophores 26 in the near-infrared (NIR) region (Figure 4D). MPLSM provides better tissue penetration and less scattering, thus providing advantages for three-dimensional and deep-tissue imaging (≥500 µm) 27, 28 (Figure 3). Moreover, MPLSM can provide metabolic information by imaging autofluorescence of nicotinamide dinucleotide (NADH) and flavin adenine dinucleotide (FAD) using endogenous fluorophores. Additionally, MPLSM allows label-free imaging by collecting second-harmonic generation (SHG) signals 29, which occur at highly ordered non-centrosymmetric structures, such as, fibrillar collagen in the TME. Third harmonic generation (THG) microscopy is also employed to image cellular membranes, lipid droplets 30 and collagen bundles or muscle fibers 31. THG occurs at structural interfaces, such as the third-order nonlinear susceptibility χ3. Unlike SHG, THG does not require a specific asymmetric structure to be imaged and is usually elicited by water-lipid and water-protein interfaces. It causes a tripling of the frequency of the excitation wavelength, whereby the combined energy of three photons is converted into one emitted photon with one-third of the excitation wavelength but triple the energy 32. Thus, THG has demonstrated excellent potential for label-free intravital imaging.

**Figure 5:**
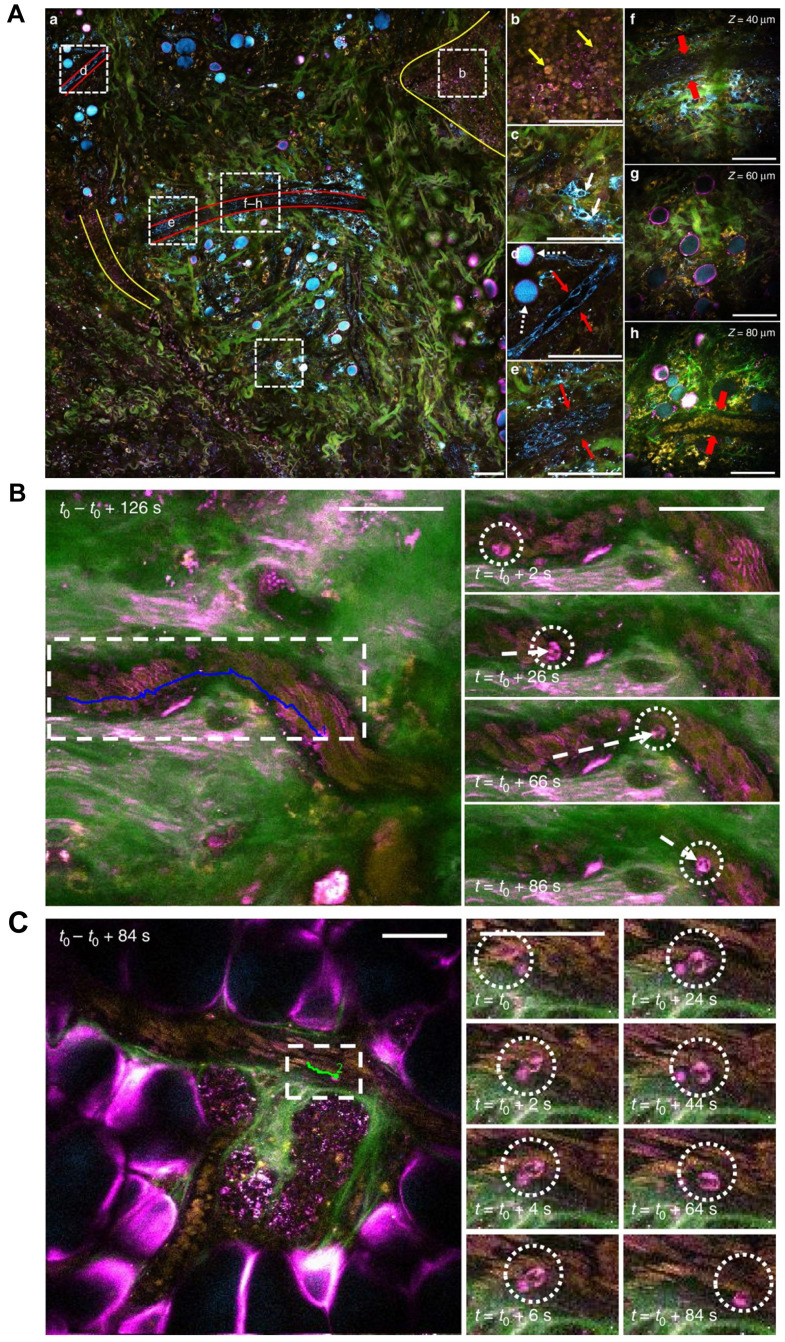
***In vivo* label-free multi-color tumor vascular imaging.** (**A**). Tumor microenvironment of a live rat by SLAM microscopy. A full large field of view (1.5 × 1.5 mm^2^) at the N-Nitroso-N-methylurea induced mammary tumor boundary is shown (**A-a**), with different regions of interest (white dashed squares) magnified in **b**-**g**. Tumor cell clusters and vasculature are highlighted with yellow and red boundaries, respectively. Green: SHG (collagen fibers), magenta: THG (interface), yellow: 2PAF (FAD), cyan: 3PAF (NADH). **(A)-b** Tumor cells (10 µm, yellow arrows) with co-localized 2PAF (FAD) and THG (interface) signals. **(A)-c** Larger (>20 µm) cells (white arrows) with strong 3PAF (NADH) signals, which are likely to be macrophages based on their irregular cell shape and oval-shaped nucleus. **(A)-d, e** Vascular endothelial cells (red arrows) and adipocytes with THG-strong boundaries and NADH-strong content inside (white dashed arrows). **(A)-f-h** Images acquired from different depths, showing a hollow vessel composed of a layer of endothelial cells, without red blood cells (**f**), and a mature vessel filled with red blood cells (**h**, contrast is enhanced to show blood cells in deeper layers). Scale bar: 100 µm. **(B, C).** Two modes of leukocyte arrest captured by SLAM microscopy. A multi-nucleated neutrophil slowly rolling along the vessel wall (**B**) and immediate arrest of a leukocyte with a sudden halt in the movement, followed by adhesion and crawling (**C**). Scale bar: 50 µm. Modified from 35 with permission, 2018 Springer Nature.

MPLSM was first used in *in vivo* imaging and quantification of tumor angiogenesis in 2001 by Brown EB* et al.* 33. Since then, multiphoton microscopy has been widely utilized in tumor vascular imaging 20, 34. A recent development in this field is simultaneous label-free autofluorescence-multiharmonic (SLAM) microscopy, developed by You S *et al*. It features fast epi-detection of NADH from three-photon autofluorescence (3PAF) and FAD from two-photon autofluorescence (2PAF), combined with non-centrosymmetric structures from SHG and tissue interface features from THG in a single-excitation source nonlinear imaging platform (with a custom-designed excitation window at 1110 nm and shaped ultrafast pulses at 10 MHz) (Figure 5A) 35. By using SLAM microscopy, they visualized the leukocyte recruitment cascade without labeling in a rat mammary tumor model. Two modes of leukocyte arrest in the tumor vasculature, slow rolling (Figure 5B) and immediate arrest (Figure 5C), were detected. Another two-photon phosphorescence lifetime microscopy (2PLM) can measure the oxygen partial pressure (PO_2_) and blood flow using specific molecular probes 36, 37. A recent study further demonstrated the strength of MPLSM for stain-free histopathology by integrating 2PAF, 3PAF, SHG, THG and coherent anti-Stokes Raman scattering (CARS) or stimulated Raman scattering (SRS), enabling the detection of a variety of vital events in carcinogenesis including tumor cell migration and angiogenesis 38.

Motion artifacts induced by heartbeats and respiration in the live animal are significant challenges to intravital imaging. One solution is to increase the scanning rate, then collect only those images obtained between the movements, a method of retroactive gating. The SDCM 39 has an opaque disk with hundreds of pinholes arranged in spirals that rotates at high speeds with extremely high scanning speed and low photodamage (up to ~1000 fps) (Figure 4A, right panel) 40. Therefore, it is a valuable tool for recording rapid live events without motion-derived artifacts. Another solution is to correct the drift during or post imaging. A real-time tissue-drift correction system using graphics processing unit (GPU) computing. that can correct specimen drift along all axes at a speed of 150 ms per stack during scanning on the 2PE has been developed 41. A new open-source software tool Galene can correct small movements in images collected by fluorescence lifetime imaging microscopy (FLIM) that require long acquisition times 42. Recently, adaptive optics, a technology that improves the image quality of fluorescence microscopy by correcting sample-induced optical aberrations, has been developed with promising results 43.

Simultaneous acquisition of tumor vessel structure and function is important for determining the metabolic state in the TME. In addition to obtaining information on NADH/FAD via 2PE/3PE, IVM methods can determine glucose uptake, mitochondrial membrane potential (MMP), and oxygen saturation (_S_O2) in cancer implants *in vivo* using fluorescence contrast agent 2- [N-(7-nitrobenz-2-oxa-1, 3-diaxol-4-yl) amino]-2-deoxyglucose (2-NBDG), tetramethyl-rhodamine ethyl ester (TMRE), and differential absorption spectra of oxygenated and deoxygenated hemoglobin, respectively 44. In metastatic 4T1 mouse breast cancers, increased glucose uptake was observed across all _S_O2 levels, indicating the "Warburg effect", and showed increased MMP relative to normal tissue. The 4T1 peritumoral areas showed a significant glycolytic shift relative to the tumor regions. In contrast, non-metastatic 67NR and 4T07 mouse breast cancers displayed increased MMP but comparable glucose uptake relative to normal tissue.

**Figure 6:**
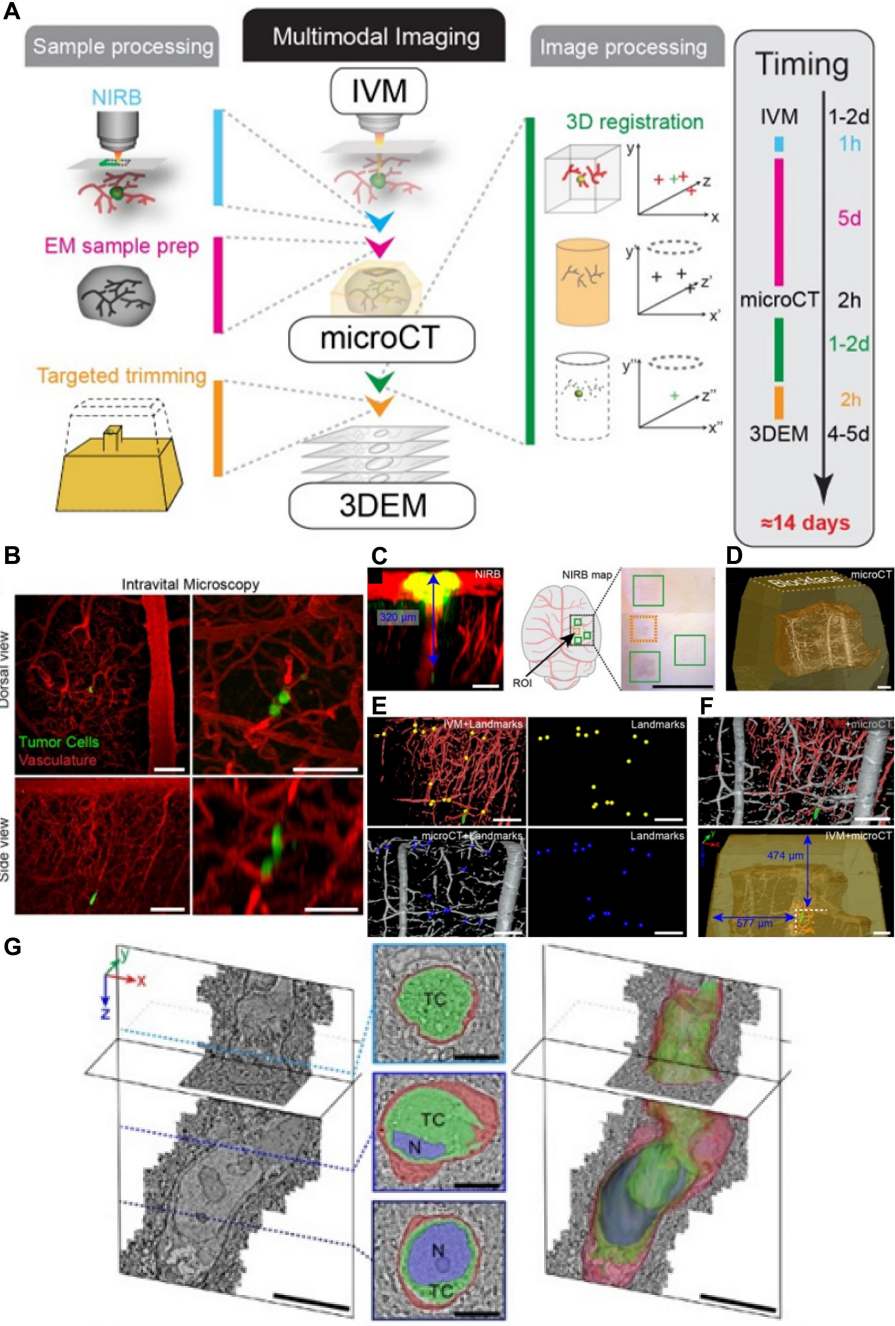
** Multimodal vascular imaging combining IVM, micro-CT, and 3D-EM. (A).** Workflow for multimodal correlative microscopy. First, the event of interest is captured with IVM (1-2 days), and an ROI is marked at the tissue surface with near-infrared branding (NIRB) (1 hour). A biopsy containing the ROI is dissected and resin-embedded for EM analysis (1+4 days), and then imaged with micro-CT (2 hours). The IVM imaged volume is registered to the micro-CT volume using Amira software (1-2 days), which allows determination of the position of the resin-embedded ROI relative to the surface of the block. The resin block is trimmed to expose the tumor cell for EM imaging (2 hours). Finally, 3D-EM of the ROI is performed (4-5 days). **(B-F).** IVM and micro-CT imaging of tumor cells in the mouse brain cortex blood capillary after intracardiac injection of the cells, followed by 3D registration. **(B).** IVM analysis of an arrested tumor cell (green) in the brain cortex vasculature (red). Dorsal views (*z*-projection, large field of view, top panels) and side views (*x*,* z*-projection, small field of view, bottom panels) of the ROI, 2 days (left panels) and 3 days (right panels) post tumor cell injection. Scale bars: 100 μm in left panels, 50 μm in right panels. **(C).** After IVM acquisition and perfusion fixation, the ROI is marked with NIRB on the brain surface, producing autofluorescence (yellow) in both the green and red channels. The *x*,* z* projection (left) shows the distance (blue arrow) between the tumor cell (green, lower in the panel) and the NIRB landmark at the surface. A cartoon and an image of the mouse brain show the NIRB landmark (orange box) positioned above the ROI and three additional reference marks (green boxes) created to facilitate targeting of the ROI when dissecting the biopsies. Scale bars: 100 μm (left panels), 1 mm (right panels). **(D).** The micro-CT shows the tissue biopsy (brown) within the resin block (yellow) and the vessels (gray). Scale bar: 100 μm. **(E).** 3D registration of the vasculature as segmented from the IVM (top) and micro-CT (bottom). Corresponding points are marked in the IVM (yellow spheres) and micro-CT (blue spheres) volumes. Scale bars: 100 μm. **(F).** Based on the marks generated in **E,** the IVM volume is registered into the micro-CT data with Amira software (top), enabling precise determination of the tumor cell (green) position within the resin block and relative to its surface (bottom). Scale bars: 100 μm. **(G).** Focused ion beam scanning EM imaging of a tumor cell arrested in the vasculature (z-stack,6350 frames, 8 nm isotropic voxel size). Image frames were segmented to show the tumor cell (TC, green), its nucleus (N, blue) and the capillary's endothelial cells and basal lamina (red). Scale bars: 10 μm (3D models), 5 μm (segmented sections). (Modified from 45 with permission 2016 Company of Biologists Ltd).

No single imaging technique can visualize tumor blood vessels at multiscale (Table 1), and this has led to the development of multimodal imaging based on several intravital imaging techniques. Karreman MA *et al.* proposed a multimodal correlative approach combining IVM, micro-CT, and three-dimensional (3D)-electron microscopy (EM) (Figure 6). They captured single tumor cells (JIMT1 GFP-labeled HER2-positive human breast cancer) in the vasculature (labeled with 500 kDa TRITC-dextran) of the cerebral cortex and subcutaneous tumors, providing unique insights into metastatic events. With this method, accurate (<5 µm) correlation of functional imaging to the ultrastructural analysis of single cells is possible in a relevant microenvironment 45.

#### IVM: Light-sheet fluorescence microscopy and scanning light-field microscopy

In addition to laser scanning confocal microscopy, MPLSM, and SDCM, light-sheet fluorescence microscopy (LSFM) 46 is emerging as a promising tool for imaging tumor vessels. LSFM uses a thin sheet of light to excite only fluorophores within the focal plane (Figure 4B). As such, LSFM has a true optical sectioning capability and, hence, provides excellent axial resolution while minimizing the photobleaching and phototoxicity to a fraction of the sample. The average spatial resolution is ~230 nm in the lateral dimension and ~450-600 nm in the axial dimension. LSFM is commonly used for long-term 3D observations of live biological specimens such as Caenorhabditis elegans and zebrafish 47. Voleti V* et al.* developed an improved swept, confocally aligned planar excitation (SCAPE) microscope with light-sheet illumination that can achieve high-resolution volumetric imaging at over 300 volumes per second with over 1.2 GHz pixel rates. They successfully analyzed real-time blood flow and calcium dynamics in the beating heart of transgenic zebrafish expressing enhanced green fluorescent protein (EGFP) in endothelial cells and DsRed in red blood cells (RBCs) (Tg(flk1:EGFP)s843; Tg(gata1:DsRed)sd2) 48. LSFM has been combined with tissue-clearing techniques for 3D organ imaging. Applications include exploring tumor tissue complexity 49, the brain microvasculature and routes of glioblastoma cell invasion 50 and heterogeneity of macrophage infiltration and therapeutic response in lung carcinoma 51. LSFM has been recently reviewed 52.

**Figure 7:**
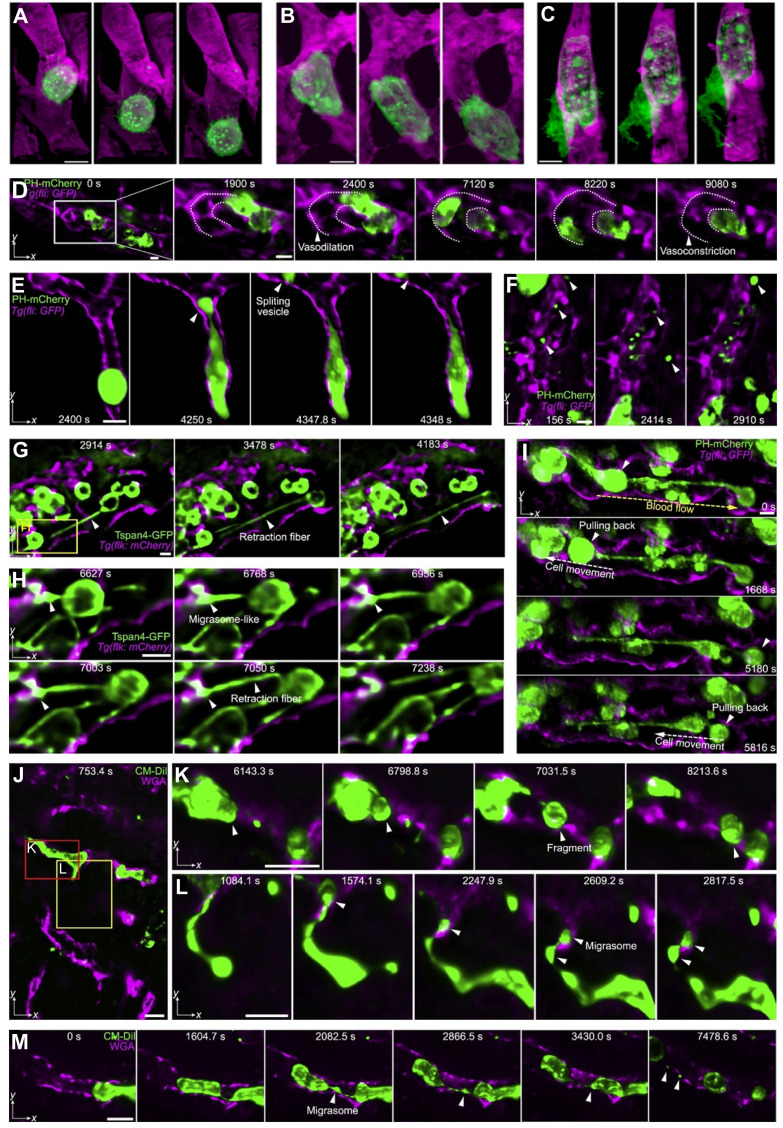
** Subcellular intravital imaging of tumor cell migration and membrane dynamics in zebrafish and mouse models.** (**A-C**). MDA-MB-231 human breast cancer cells (mCherry-CAAX, green) were intravenously injected into a transgenic zebrafish embryo 48 hpf (endothelial cell kdrl: gfp, magenda). The cells are observed rolling in a vessel (A) or crawling along the endothelium (**B**). A cancer cell in transendothelial migration shows an increasingly complex morphology over time (**C**). Scale bars, 10 µm. Adapted from 53 with permission 2018 The American Association for the Advancement of Science. (**D-I**) MDA-MB-231 cells injected pericardially into blood vessels of a transgenic zebrafish larvae [Tg (fli: GFP), magenta] are observed. (**D**) Maximum intensity projections show vasodilation as a cancer cell (PH-mCherry, green) approaches a turn in the vessel and vasoconstriction is seen as the cell exits the field of view (dashed lines: vessel contour). (**E**) A tumor cell splits a large vesicle (arrowhead) when trapped in a small-bore vessel. (**F**) Various vesicles are observed flowing in the vessels. (**G** and **H**) Cancer cells (Tspan4-GFP, green) generate retraction fibers and migrasome-like vesicles when dissociating from multicellular clumps under flow shear stress. (**I**) Cancer cells (PH-mCherry, arrowheads) are pulled back by retraction fibers even against the blood flow. The direction of blood flow (yellow dashed arrow) and that of cell movement (white dashed arrow) are indicated. (**J-M**) HeLa human cervical cancer cells (membrane dye CM-DiI, green) migrate in mouse liver vasculatures following a splenic injection. The vessels are labeled by intravenously injected AF647-wheat germ agglutinin (magenta). HeLa cells split off large fragments when trapped in the small vessels (**K**) and generate retraction fibers and migrasomes during migration (**L** and **M**). Scale bar, 10 μm. Adapted from 54 with permission, 2021 Elsevier.

Modifications to LSFM have further improved its utility. Liu TL *et al.* combined non-invasive lattice light-sheet microscopy with aberration-correcting adaptive optics to study a variety of delicate subcellular events *in vivo*, including organelle remodeling during mitosis. Various tumor cell motions within blood vessels of zebrafish embryos have also been studied: tumor cells exhibit rolling while extending long, adhesive microvilli and crawling while conforming to the shape of a vessel, and, finally, transendothelial extravasation (Figure 7A-C) 53. However, this technique is applicable only to a small field of view imaging, and tiling the corrective adaptive optics restricts overall speed over a large volume.

Long-term, high-speed imaging at subcellular resolution and low photon doses remains a challenge in mammals. To overcome these barriers, Wu J *et al.* proposed a computational imaging framework, termed digital adaptive optics scanning light-field mutual iterative tomography (DAOSLIMIT) 54. It features high-speed, high-resolution 3D imaging, tiled wavefront correction, and low phototoxicity. In contrast to LSFM which tomographically scans through the volume, plane by plane, light-field microscopy acquires the entire volume simultaneously using an extended depth of field (Figure 4C). Volumetric imaging of 225 × 225 × 16 μm^3^, with a resolution of up to 220 nm laterally and 400 nm axially, is obtained at the millisecond scale resulting in hundreds of thousands of data points. With DAOSLIMIT, Wu J *et al* studied the dynamics of cancer cell migration by injecting human breast cancer cells expressing PH-mCherry into the vasculature of zebrafish expressing EGFP in endothelial cells [Tg(fli:eGFP)] 54. Vasodilation occurs as a cancer cell approaches a vessel turn, followed by vasoconstriction as the cell floats away (Figure 7D). Notably, some cancer cells are trapped in small-bore microvessels, and large internal vesicles can be seen to split and be released under the stress of intravascular flow (Figure 7E-F). These observations raise some interesting possibilities: while cancer cells may reduce their cell-body volume to escape from small-bore microvessels, these “splitting vesicles” may deliver signals to distant organs promoting further metastases. Similarly, circulating tumor cells-derived microparticles have been reported to migrate along the lung vasculature and are mainly taken up by interstitial myeloid cells, contributing to metastatic seeding 55. Another interesting observation is the generation of retraction fibers and migrasome-like vesicle structures when multicellular clumps of cells are retained in vessels (Figures 7G-H).

Long-term imaging showed that some of the escaping circulating tumor cells were pulled back to the multicellular clumps by retraction fibers even against strong blood flow (Figure 7I), which suggests that retraction fibers help maintain circulating tumor cells as clusters. The investigators found similar phenomena in mammals as well. HeLa cells trapped in small vessels demonstrated split large vesicles and cell fragments within mouse livers (Figure 7J-K) and also generate retraction fibers and migrasomes during migrations along the vasculature (Figures 7L-M). These observations reveal unexpected and complex behaviors of circulating cancer cells and highlight the importance of DAOSLIMIT for understanding this process more thoroughly.

#### IVM in the second near-infrared window

Light scattering by biologic tissues limits the penetration of light depth in tissues of live mammals *in vivo*. A practical approach to reduce light scattering and increase imaging depth is to extend the excitation and emission wavelengths to the second near-infrared window (NIR-II: >1,000 nm). The NIR spectrum region is divided into three channels: NIR-I (700-900 nm), NIR-IIa (1,300-1,400 nm), and NIR-IIb (1,500-1,700 nm) channels. Visualizing tumor vasculatures via a NIR-II imaging system relies on a NIR-II contrast agent. In the past 15 years, NIR-II contrast agents have significantly advanced 56. Examples include, inorganic single-walled carbon nanotubes (SWNTs) 57, 58, quantum dots 59, rare-earth doped down-conversion nanoparticles 60, and organic NIR-II molecules or protein complex 61, 62, such as aggregation-induced emission (AIE) luminogens (AIEgens) 63.

Wide-field fluorescence imaging in the NIR-II window combined with one or more of those contrast agents can achieve 1~4 mm of imaging depth but remains limited in resolution. Various NIR-II microscopies have been developed recently with high-resolution and/or 3D mapping capability. NIR-II spinning-disc confocal microscopy 64, has a lateral resolution of 0.5 ± 0.1 µm and an axial resolution of 0.6 ± 0.1 µm. NIR-II light-sheet microscopy (LSM) with excitation and emission of up to approximately 1,320 nm and 1,700 nm, respectively, has a penetration depth of up to 750 μm, lateral resolution of 0.9-1.6 µm and an axial resolution of 2.6-4.4 µm. In addition to imaging tumor microcirculation with NIR-II LSM, 3D molecular imaging of immune checkpoint proteins has been performed at the cellular scale in live animals 65. This imaging system was extended to structured-illumination light-sheet microscopy (NIR-II SIM) with excitation and emission wavelengths in the NIR-IIb window 66. The integration of structured-illumination into NIR-II light-sheet microscopy diminished out-of-focus background, improving the spatial resolution by 1.7 times and facilitating long-term *in vivo* imaging of cell behavior in intact tissues without surgery. Moreover, this team also developed superconducting nanowires with single-photon counting detectors and quantum dots for longer than 1,700 nm to conduct confocal fluorescence microscopy in intact rodents 67.

#### Quantification of hemodynamics using IVM

In addition to the development of microscopy, advanced quantitative methodologies for measuring blood flow in tissues, including tumors, is important. Kamoun WS *et al.* developed two MPLSM-based methods to simultaneously quantify blood flow (i.e., velocity, flux, hematocrit, and shear rate) in complicated blood vessel networks at single-capillary resolution *in vivo* 68. One is called residence timeline scanning (RTLS), which allows direct flow velocity analysis by scanning the line at an arbitrary angle to the vessel. The other is relative velocity field scanning (RVFS), which is a full-field method that allows simultaneous analysis of most vessels in a field of view by deconvolving the image distortion produced by the moving cells relative to the moving laser scans. Honkura N *et al.* developed a time-lapse intravital imaging-based image analysis tool, RVDM (relative velocity, direction, and morphology), to monitor rapid changes in specific types of vessels 69. RVDM relies on the laser scan speed, relative velocity, and direction of blood flow, to produce parametric images of RBCs in vessels. It allows the identification of vessel categories by their RBC velocity dynamics. By using RVDM in transgenic mice expressing GFP in the endothelial cells under a tight junction marker Claudin5 (Cldn5) promoter [*Cldn5*(BAC)-GFP), they revealed that capillaries and postcapillary venules in normal mouse skin, with no or low Cldn5 expression, respond to VEGFA with rapid changes in blood flow, vessel tone, and leakage. This approach could also be used in distinguishing heterogeneous tumor vessels in future.

Hypoxia in solid tumors can be a limiting factor for therapeutic efficacy. Hypoxic heterogeneity is considered to arise from abnormal tumor vasculature, but the precise mechanisms are not well understood. Bernabeu MO *et al.* introduced a metric to characterize tumor vasculature (mean vessel length-to-diameter ratio, λ) and demonstrated how it predicts tissue-oxygen heterogeneity, based on MPLSM imaging 70. RBC transport plays a role in establishing oxygen heterogeneity in tumor tissue, and λ could be used to monitor the efficacy of antiangiogenic agents and provide a proxy measure of perfusion and oxygenation in the tumor. Moreover, Enjalbert R *et al.* proposed a computational model to investigate the impact of vessel compression on RBC dynamics in tumor vascular networks 71. Vessel compression alters RBC partitioning at bifurcations in a hematocrit-dependent and flow rate-independent manner. These findings contribute to hemodilution and partitioning of RBCs within tumor vascular networks resulting in regional hypoxia that may be reversible following pharmacological decompression.

#### Other techniques: Photoacoustic imaging

Photoacoustic imaging (PAI) is a non-invasive imaging technique that utilizes non-ionizing laser pulses to induce ultrasound pulses that can be detected with an ultrasound transducer. The light pulse absorption by molecules in tissues creates a thermal pressure wave that propagates ultrasonic waves, which are detectable with ultrasound transducers (light in-sound out). In conventional ultrasound, the sound is produced at the transducer, and the reflected sound is detected again at the transducer (sound in-sound out). There are three major types of PAI based on resolution and image depth: optical-resolution photoacoustic microscopy (OR-PAM, resolution: 0.2-10 µm, depth: 1-2 mm), acoustic-resolution photoacoustic microscopy (AR-PAM, resolution: 15-50 µm, depth: 3-10 mm) and photoacoustic computed tomography (PAT, resolution: 100-500 µm, depth: 10-100 mm) 72. Therefore, PAI encompasses a range of spatial resolution that at one end can detect organelles while at the other end can image whole animals or humans 73, 74 (Figure 3). PAI has developed rapidly in the last decades, with innovations such as fast PAI 75, high-throughput PAI 76, deep blood oxygenation imaging (in whole tissue) 77 and multi-modal imaging (photoacoustic and SDCM or 2P and SHG microscopy) *in vivo* 78, 79*.*

Optical absorption reflects endogenous total hemoglobin concentration (THb = Hb + HbO2) and oxygen saturation (sO2 = HbO2 / THb), making PAI a useful tool to monitor vasculature changes, such as diameter, density, and tortuosity, in early tumors and oxygen saturation. Regional hypoxia can be demonstrated within solid tumors using Thb and blood _S_O2 PAI. This has been confirmed in several tumor models, such as melanoma metastasis in brain 80 and xenograft and orthotopic breast cancer models 81, 82. In general, Thb tends to be lower in the tumor core than in the periphery, consistent with poor central perfusion and high oxygen consumption. By changing the gas delivery to the mouse from room air to 100% oxygen (i.e., oxygen challenge) and measuring the induced change in sO2, an index of perfusion can be established 83, 84. For example, a gas challenge on mice bearing subcutaneous PC3 or LNCaP human prostate cancers demonstrated smaller responding areas in the former tumor than the latter, with a high disparity between rim (rapid change) and core (no dynamic response), consistent with *ex vivo* findings of hypoxic and necrotic core tissues surrounded by a well perfused vasculature rim.

Methods to enhance signal-to-noise ratios in PAI have been explored. Genetic engineering of cells to express photoacoustic contrast is an attractive method to selectively label cells of interest to study their biological behaviors *in vivo*. For instance, cells transduced with a tryrosinase produce an absorbing pigment eumelanin that creates a strong photoacoustic contrast. Tyrosinase-expressing human tumor xenograft is detectable at nearly 10 mm depth with a spatial resolution below 100 μm in mice, enabling documentation of their growth and surrounding vasculatures 85. Silver (Ag) nanoplates also have the potential as a PAI contrast agent with strong absorption for quantitative image-based evaluation of tumor vasculature and heterogeneity after intravenous infusion 86.

#### Other techniques: Optical coherence tomography

Optical coherence tomography (OCT) has made great strides in the last 30 years 87. OCT is a non-invasive, interferometry-based technique that generates cross-sectional images of tissue by detecting the back-scattered photons with NIR low-coherence light 88. Similar to ultrasound imaging in which sound pulse reflections (echoes) are measured to create an image, OCT uses the low coherence properties of a broadband light source, and light reflections which are measured with a Michelson interferometer. OCT is commonly used as a label-free technique that enables anatomical and physiological imaging of tumor blood vessels. Compared with IVM, OCT has deeper tissue penetration (2-3 mm), a wider field of view, and higher imaging speed, but suffers from lower resolution (<10 μm) (Figure 3). Vascular OCT imaging has been widely used in both preclinical and clinical studies 89, 90. It has been employed to visualize blood vessels in human malignant melanoma and normal skin 91, identify specific tumor vasculature subtypes in human choroidal osteoma 92, and observe vasculature response to vascular-targeted photodynamic therapy 93 and photoimmunotherapy 94. Research advances in vascular OCT imaging have been reviewed elsewhere 95, 96. Multimodal OCT addresses the limitations of OCT by combining it with other imaging modalities. For instance, in a study using an orthotopic melanoma model, murine tumor capillaries were visualized at various resolutions, and vessel density quantification was compared across different modalities, including MRI, PET, CT, ultrasound, OCT, and high-resolution episcopic microscopy 97.

### Recent advances in animal window models

In addition to the imaging techniques discussed above, achieving clear visualization of tumor vasculature at high resolution relies on the selection of animal window models and their technical advancements.

#### Animal window models and surgical preparation

Since the early intravital imaging of lymph nodes 20 years ago 98, 99, various rodent window techniques have been introduced to visualize blood vessels, from the superficial skin to internal organs (Supporting Table 1). The dorsal window chamber (DSWC) is created by stretching skin tissue over a ring, whereupon a tumor cell injection or tumor tissue implantation is performed on the dorsal skin in order to observe subcutaneous tumor progression and vasculature changes 100. Smaller and lighter DSWC minimize animal burden 101, 102. Another type of superficial window model is the mammary fat pad-based window models (MWCs), primarily utilized for visualizing the blood vessels in orthotopic breast cancer models or studying the metastasis behavior of tumor cells 103. These windows are typically installed once the tumor has formed. Both DSWCs and MWCs are compatible with MRI and other imaging modalities 104, 105. Furthermore, a recent development involves a flexible and sutureless silicon window that enables visualization of muscle tissue and orthotopic breast cancer 106. Bone windows have been created for imaging brain tumor vasculature 107 and bone marrow 108, 109. The cranial window 107 is mainly used for cerebral vessel imaging while the femur window 110, 111 is used for bone marrow imaging. Either method can be used for tracking single cell extravasation as well as visualizing both tumor and endothelial dynamics over a long time period. The cranial window to visualize tumor blood vessels in the brain utilizes either thinned skull transcranial windows, reinforced thinned skull windows, transcranial windows, implanted windows or implanted prism, or lens windows 112. Cranial windows can be used in combination with other imaging modalities, for example, ultrasonography and MPSLM/OCT 113, MRI, MPLSM and CT 114, and MPLSM and PET 112 imaging.

Imaging internal organs, especially within the thoracic or abdominal cavities, poses significant challenges due to their inaccessibility and the continuous motion of these organs. To enable high-resolution imaging of blood vessels in the lungs, several vacuum-stabilized lung windows have been developed. These windows can be used for extended periods, typically lasting several hours 115, 116. Additionally, a long-term murine lung window has been introduced, allowing for the visualization of cancer metastasis through the blood vessels over a span of several weeks 117. Similarly, intravital vascular imaging in the liver involves both simple non-survival models (that can last 6 hours 118, 119) and longitudinal observation models (4-5 weeks) that utilize titanium abdominal windows 120 (Supporting Table 1). These abdominal window techniques are applicable to imaging other organs, such as the spleen, kidney, intestines, and pancreas 120, 121. For example, they have been used to observe insulin secretion from pancreatic tail 122, and to characterize abnormal tumor blood vessels in various pancreatic cancers, including spontaneous transgenic tumors and orthotopically inoculated cancers, by comparing them to the normal pancreatic vasculature 123. Abdominal windows are also compatible with multiple-modality imaging, such as combing MPLSM with laser speckles imaging, or MPLSM with MRI 122, 123. Furthermore, the use of window models has been extended to evaluate the normal reproductive system, including the ovary 124 as well as recently implanted embryos 125.

#### Labeling methods

Visualization of vessels mostly relies on some form of blood vessel tagging. Two types of vessel detection methods are used in intravital vascular imaging: One uses transgenic animals, in which endothelial cells exhibit a specific fluorescent signal. For example, in the endothelial nitric oxide synthase transgenic (eNOS)tag-GFP mice 102, GFP positive cells represent the endothelial cells, while in the VeCad-tdTomato transgenic mice 117, TdTomato fluorescence protein is expressed in the endothelial cells. Another approach is to use exogenous labeling such as a dye conjugated to albumin that has large molecular mass to remain intravascular. Such agents can be intravenously injected, enhancing the blood vessel. Alternatively, a dye-conjugated antibody specific for an endothelial cell marker can be infused to tag the endothelial cells lining the vessels. Dextrans with various molecular weights (10, 50, 70, 155, or 2000 kDa) are used to evaluate different vascular functions and leakiness of vessels. A direct infusion of a dye DiI has been used to enhance the vessels against the background 126*.* For antibody labeling, CD31, VCAM-I and P-selectin are often used as endothelial cell biomarkers (Supporting Table 1, also reviewed in 5, 127, 128).

## Tumor Vascular Responses to Therapy

Visualization of morphological and functional changes in tumor vasculature in response to therapies can offer valuable insights into the effects and mechanism of these treatments, ultimately contributing to improved treatment outcomes. This section describes intravital imaging findings of dynamic vascular responses to different therapies and discusses new insights implicating an immune-vascular crosstalk observed in various therapies.

### Chemotherapy

Chemotherapy is a generic term for a family of cytotoxic anticancer therapies. *In vivo* image findings of drug delivery 129, 130 and drug effects 131-133 at single-cell resolution and imaging chemotherapy induced angiogenesis and “normalization” of tumor vasculatures has been reviewed extensively. Increased pericyte coverage, improved tumor vessel perfusion, reduced vascular permeability, and consequently reduced hypoxia, have been observed during tumor vascular normalization in which the neovasculature becomes more organized in response to therapy 34, 134, 135. Here we highlight recent findings of chemotherapy-induced vascular permeability changes observed by IVM.

As described in the Introduction section, the extravasation of cells and drugs can occur through either the paracellular or transcellular pathway 9. Investigations utilizing IVM have provided insights into the paracellular pathway of cell extravasation and the involvement of cells and cytokines in treatment-induced changes in permeability. For instance, in a spontaneous mouse model of breast cancer (MMTV-PyMT mice), IVM helped to demonstrate that doxorubicin treatment induces recruitment of myeloid cells expressing matrix metalloproteinase 9 (MMP9) into the tumor via the CCL2/CCR2 chemokine/chemokine receptor axis. This recruitment correlated with the degree of vascular leakage, as detected using 10 kD dextran. Interestingly, in the absence of MMP9, vascular leakage increased due to enlarged endothelial gap junctions and decreased pericyte coverage, resulting in a better tumor response to doxorubicin treatment 136.

Harney *et al.* showed that the secretion of VEGFA by perivascular TIE2^hi^ macrophages led to a local transient increase in vascular permeability, known as "bursting," which facilitated intravasation of breast cancer cells through vessels with reduced pericyte coverage and junction integrity 137. This finding supports the concept of the "tumor microenvironment of metastasis" (TMEM), which involves a perivascular TAM contacting with a mammalian-enabled (MENA)-overexpressing tumor cell and endothelial cell, creating a front for cancer cell intravasation 137. Similarly, paclitaxel treatment increased the infiltration of perivascular TIE2^hi^/VEGF^hi^ macrophages, induced TMEM-dependent transient and localized vascular permeability, and enhanced breast cancer metastasis. This observation correlated with high expression of MENA on the disseminating cancer cells 138. Other common chemotherapy treatments, such as doxorubicin/cyclophosphamide treatment, showed similar pro-metastatic changes. Conversely, inhibition of the TIE2 receptor using rebastinib or knockdown of the MENA gene reversed chemotherapy induced TMEM activity and cancer cell dissemination 138.

Interestingly, another study demonstrated tumor vascular "bursting" in the presence or absence of leukocytes in the perivascular area 139. In contrast to other studies that utilized dextran, this study used 30 nm or 70 nm nanoparticles to examine vascular permeability. It is possible that the nanoparticles themselves had effects on the endothelial barrier, leading to "bursting" even in the absence of perivascular leukocytes. Certain types of nanoparticles have been reported to induce endothelial leakiness by disrupting VE-cadherin-VE-cadherin homophilic interactions at the cell-cell interface, which can facilitate cancer cell intravasation and extravasation 140.

Additionally, increased tumor vessel permeability and drug delivery via the paracellular pathway have been achieved by intraperitoneal injection of a transforming growth factor β (TGF-β) inhibitor, which induces defects in pericyte coverage 141, or by using doxorubicin-containing MMP2-responsive nanoparticles that release an anti-platelet antibody, leading to local depletion of tumor-associated platelets adhering to the vessel wall 142.

The transcellular pathway can also mediate changes in vascular permeability, influencing drug delivery. This active transcytosis can occur through caveolae or vesicula-vacuolar organelles (VVOs). Caveolin-1 is critical for caveolae formation and caveolae-mediated transport, while inflammatory mediators or VEGF can induce VVOs involved in the extravasation of macromolecules 9, 143. For example, IVM and TEM have documented an active transcytosis-mediated enhancement of permeability as the primary route for transporting certain nanoparticles 11. Interestingly, neither knocking out caveolin-1 (thus lacking caveolae) nor vessel normalization using a VEGFR-2 blocking monoclonal antibody (DC101) had an impact on this transcytosis-dependent enhanced drug delivery. Further investigations are required to better understand the fundamental mechanism of the transcytosis pathway.

One strategy to induce caveolae-mediated transcytosis is the cationization of drug formulation at the luminal endothelial cell surface. This mechanism has been utilized to improve tumor penetration of camptothecin (CPT). Conjugation of CPT to a newly designed membrane γ-glutamyl transpeptidase-activatable polymer led to enhanced treatment efficacy compared to CPT-11 alone 144. Similarly, conjugation of the small drug 7-ethyl-10-hydroxycamptothecin (SN38) to a poly(2-(N-oxide-N,N-diethylamino)ethyl methacrylate) (OPDEA)-based polymer facilitated tumor penetration via transcytosis. Treatment with this agent resulted in the eradication of tumor implants and patient-derived tumor xenografts in mice 145.

In contrast to the studies indicating improved drug delivery via permeability enhancement, several studies using acute myeloid leukemia (AML) models demonstrated that decreased vascular permeability benefited the treatment outcome. MPLSM functional imaging in a skull window model of AML engraftment indicated an association of AML with vascular abnormalities. The inability to restore normal vasculature following induction chemotherapy has been linked to a poor prognosis, likely attributed to excessive nitric oxide (NO) production. Importantly, inhibition of NO production improves treatment response to arabinosylcytosine *in vivo* 146. A similar skull window model demonstrated that MMP inhibition reduced vascular permeability, suppressing AML infiltration and growth 147.

Other examples demonstrate the value of intravital microvascular imaging in understanding the impact of therapies on tumor vasculature. For instance, fibroblast growth factor 9 was revealed to normalize tumor blood vessel structure for the vessel hierarchy and vasoreactivity, reducing tumor core hypoxia and VEGF-A expression 148. Angiopoietin-4 increased vessel permeability by 4.5-fold and promoted lymphatic dilation and activation of TIE2-dependent signaling 149. EphB4 overexpression resulted in resistance to sunitinib treatment in SF126 glioma subcutaneous and orthotopic models 150. These chemotherapy-induced vascular permeability changes observed using IVM are summarized in Table 2.

### Radiation therapy

The effects of radiation therapy (RT) on blood vessels highly depend on the radiation dose, timing, fraction size, as well as the type, location, and stage of the cancer 161-163. Single, high-dose RT (> 10 Gy) has been shown to induce endothelial cell apoptosis 164, 165 by upregulation of ALK5 and sphingomyelinase 162, wherein vessels regress and blood flow decreases 166. Eventually, new vessels are formed via the recruitment of endothelial progenitor cells and improvement of pericyte coverage of the vessel wall, a process thought to be mediated by the CXCL12/CXCR4 chemokines system and PDGF-B/PDGFR-β signaling 167, 168. In contrast to high single-dose irradiation, fractionated low-dose irradiation exerts a proangiogenic effect on the vasculature by upregulating the NO pathway 169 and angiostimulatory growth factors, such as VEGF and placental growth factor, eventually inducing vascular growth and increased tissue perfusion 170.

A longitudinal investigation of human pancreatic tumor xenografts has been conducted to assess their response to different doses of RT (10, 20, and 30 Gy). This study utilized a swept-source OCT system in conjunction with a dorsal window chamber 171. The findings revealed that immediately after irradiation, poorly vascularized and low-oxygen areas within the tumor increased, reaching their peak between 2-3 weeks post-irradiation. Subsequently, these areas returned to pre-irradiation levels in approximately 3 weeks. Compared to the unirradiated controls, this effect was more significant with doses of 30 Gy and 20 Gy, but not with 10 Gy irradiation. The study provides valuable insights into the temporal vascular response to different RT doses, which could contribute to predicting RT efficacy in future applications.

Additionally, the effects of RT on tumor blood vessel permeability are time dependent. Shortly after irradiation, vascular permeability may increase because of the increased production of cytokines like VEGF. However, over time, vessel regression occurs, leading to reduced vascular permeability and a decrease in the total vascular surface area available for fluid and nutrient exchange 161. Intravital imaging has proven valuable in elucidating RT-induced permeability changes and their underlying mechanisms. For example, using confocal microscopy in a dorsal window chamber with HT1080 human fibrosarcoma, a study examined the effects of a single low dose (5 Gy) irradiation. The findings revealed that irradiation induced a transient, dynamic, and localized vascular "bursting" phenomenon, leading to increased vessel volume, permeability, and tortuosity, thereby enhancing drug delivery 151. Additionally, the study identified perivascular TAMs as being responsible for these vascular bursts and the subsequent enhanced drug delivery.

### Immunotherapy

Cancer immunotherapy has revolutionized cancer treatment and was named the 2013 “Breakthrough of the Year” by the journal Science 172. Immunotherapy is a broad category of therapy and includes therapies that use immune checkpoint inhibitors (ICIs), cytokines, adoptive cell transfer, targeted monoclonal antibodies, oncolytic viruses, and cancer vaccines 173-175. This subsection illustrates recent intravital imaging observations in dynamic vascular responses to immunotherapy.

Programmed-death ligand 1 (PD-L1) is an immune checkpoint protein expressed by certain tumor cells that binds to programmed cell death protein 1 (PD-1) expressed on T cells, thereby inactivating T cells and evading immune surveillance. PD-L1 blocking antibodies, therefore, can activate the immune system and treat cancer 176. Non-invasive *in vivo* 3D three-plex molecular imaging of a PD-L1-expressing CT26 mouse colon carcinoma was recently performed via NIR-II LSM 65. In this study, the tumor cells, T cells, and vessels were labeled *in vivo* by intravenously injected Erbium-based rare-earth nanoparticles (ErNPs) conjugated to anti-PD-L1 antibody, PEGylated PbS/CdS core/shell quantum dot (CSQD) conjugated to anti-PD-1 antibody, and p-FE (comprised of organic dyes trapped in amphiphilic polymeric micelles approximately 12 nm in size), respectively. PD-1^+^ T cells extravasating from vessels as well as surrounding PD-L1^+^ tumor cells were visualized, representing the first step in cancer cell-killing. This non-invasive imaging approach could be used in longitudinal studies to understand the mechanisms of therapy resistance. A possible way to overcome this problem has been reported based on an interesting observation in a dorsal window chamber model by Arlauckas SP and collaborators 177. They tracked Alexa Fluor 647 conjugated anti-PD-1 antibody infused to label T cells in DPE-GFP/GREAT mice bearing MC38-H2B-mApple tumors. The anti-PD-1 antibody rapidly perfused tumor vessels spread into the tumor interstitium and bound to T cells as early as 5 minutes after injection. However, within minutes, the anti-PD-1 antibodies were transferred to neighboring PD-1 negative TAMs (labeled *in vivo* with phagocytosis of Pacific Blue-dextran nanoparticles) via Fc binding to the Fc-gamma (γ) receptors (FcγRs) expressed on TAMs, compromising the efficacy of PD-1 therapy. *In vivo* blockade of FcγRs before the anti-PD-1 antibody administration substantially improved the antibody binding to tumor-infiltrating CD8 T cells and enhanced therapeutic response.

The role of cytokines in immunotherapy response can be explored with IVM and tumor windows. Cytokines are soluble proteins that mediate cell-to-cell communication. Recombinant interferon (IFN) alpha (IFN-α) and interleukin (IL)-2 have been approved for the treatment of several malignancies 178. Intravital imaging demonstrated that the cytokine effects on the tumor blood vessels are dose and time dependent. For example, IFN-γ was reported to induce regression of tumor vasculature between 24 and 96 hours after cytokine injection, resulting in the arrest of blood flow and subsequent collapse of tumors. In contrast, TNF was reported to dilate the blood vessels 24 hours after injection and induced a vascular “burst” at 36 hours 152. In another study, administration of IL-1β at a concentration of 2 μg/ml, but not 1 µg/ml, to mice bearing intracranial M6 glioma showed increased tumor blood vessel permeability 154.

Although promising, responses of cancer patients to immunotherapy are unpredictable and only a minority of patients respond to a single therapy. Therefore, combination therapies are being explored to improve patient outcomes. Combinations of immunotherapy with antiangiogenics, such as VEGF and angiopoietin 2, have been reviewed previously 179-182. Another approach is to combine immunotherapy with RT. Longitudinal *in vivo* imaging and functional analysis revealed that 85% and 65% of T cells survive by fractionated IR (5 doses of 1.8 Gy with 24 hour-intervals) and stereotactic body radiotherapy (SBRT, 20 Gy single dose), respectively, and show increased motility and higher production of IFN-γ compared with T cells from unirradiated tumors 183. Intratumoral T cells in irradiated tumors appear to mediate tumor even without a fresh influx of unirradiated T cells. This study suggests new pathways for optimizing RT/immunotherapy combinations by uncovering a role for radiation-resistant intratumoral T cells. Moreover, RT facilitated rapid CAR T-cell extravasation and expansion within the tumor microenvironment, possibly due to increased vessel permeability, highlighting potential opportunities to improve adoptive T-cell therapy of GBM using concurrent RT 184. Thus, combination immunotherapy shows some promise but the effects on the vessels can be difficult to predict.

### Photodynamic therapy (PDT), photothermal therapy (PTT) and photoimmunotherapy (PIT)

Beyond immunotherapy, there are several other promising light-based anti-cancer therapies, such as photodynamic therapy (PDT), photothermal therapy (PTT), and photoimmunotherapy (PIT). We illustrate tumor blood vessel response to these photon-based therapies.

PDT is a treatment that uses a tumor-tropic photosensitizer that produces tissue-damaging cytotoxic reactive oxygen species upon exposure to light 185. In the 1970s, PDT was first observed to induce vasoconstriction, occlusion, and platelet aggregation, leading to reduced tissue perfusion and hyperpermeability 185. Furthermore, PDT increases vascular permeability due to the formation of endothelial intercellular gaps, which are likely induced by endothelial cell microtubule depolymerization and apoptosis 158. IVM revealed that the effect of PDT on the vasculatures was dose dependent. For example, PDT using 1.0 mg/kg of photosensitizer verteporfin was more effective in inhibiting blood perfusion via thrombus formation, while a lower dose (0.25-0.5 mg/kg) verteporfin-PDT was more potent in enhancing dextran extravasation in an orthotopic R3327-MatLyLu rat prostate tumor model 156. PDT also demonstrates vascular effects in a manner that dependents on time after the treatment. In contrast to chemotherapy or immunotherapy, vascular effect of PDT showed similar changes when using different photosensitizers in various animal models. For example, PDT using the photosensitizer MV6401 showed biphasic blood flow changes with initial vasoconstriction in mouse mammary tumor (MCaIV) 155. The acute responses were observed immediately after PDT, and longer-term responses, such as thrombus formation, were observed more than 3 hours after PDT. Similarly, F8-small immunoprotein SIP mediated PDT induced microvascular stasis and thrombosis with reduced functional vessel density and perfusion in glioma, whereas total vessel density was not altered 24 hours after the treatment. The microvascular dysfunction recovered 4 days after treatment 186. Acute vascular responses to PDT with an EGFR-targeted nanobody-photosensitizer have been evaluated by both intravital imaging using a skin-fold chamber model and dynamic contrast-enhanced MRI. Intravital imaging showed vasoconstriction and leakage in tumor and normal tissues in response to this nanobody PDT, and dynamic contrast-enhanced MRI confirmed a decrease in tumor perfusion 187. Moreover, a recent high-resolution PAI with the ability to simultaneously record oxygenation images revealed that PDT might induce ischemia-reperfusion injury and provided an important insight into the mechanism of action of PDT. It showed that PDT with padeliporfin induced a substantial and immediate occlusion of the tumor vessels followed by hemorrhage within the tissue, and eventual collapse of the entire vasculature. Surprisingly, a temporal increase in oxygenation of the entire tumor volume was observed 5 mins after PDT, indicating rapid reperfusion from outside vessels, then dropped up to 24 hours 188.

Photothermal therapy (PTT) is another light-based noninvasive and selective treatment strategy for tumors. Unlike PDT that depends on production of reactive oxygen species, PTT functions via photothermal effects induced by converting light energy of near-infrared laser irradiation into heat 189. Laser photocoagulation specifically affects the blood vessels surrounding and supplying nutrients and oxygen to the tumors and results in thermal destruction of the tumor tissue. The vascular disruption of VEGF-targeted photothermal therapy was monitored via intravital imaging in an orthotopic murine glioma model 190. Vessel density increased by 18% for the saline group but decreased by 24% for the VEGF-NS treatment group over three days. A potential disadvantage of PTT is damage to surrounding normal tissues.

Photoimmunotherapy (PIT) is a cell-specific cancer therapy based on an armed antibody conjugated with a photosensive dye that induces rapid and highly selective cancer cell necrosis and immunogenic cell death after NIR irradiation. Previous studies suggest that following PIT, there is a super-enhanced permeability and retention effect 160, which allows high concentrations of nanoparticles to accumulate in the tumor relative to pre-treatment permeability levels. Real-time hemodynamic changes were monitored during PIT by OCT. It revealed that blood velocity in peripheral tumor vessels quickly drops while the vessel lumen enlarges. Ultraslow blood velocity could contribute to the long drug circulation time within the tumor, which could cause increased tumor blood pool volume 191.

## Future Perspectives

Intravital imaging of tumor vasculature has made significant technical progress and provided a growing number of new insights in preclinical tumor models as discussed above. To enable more detailed investigation of tumor vasculatures and dissect underlying mechanism of biological events involving cells and vesicles, super-resolution intravital imaging that can reach 20-70 nm resolution is required. Such high-resolution imaging can, for example, visualize dynamic vesicle transport *in vivo* and help in understanding the mechanism and regulation of active transcytosis or transport through the endothelial gaps. The knowledge obtained from these fundamental studies would lead to improved cancer treatments.

**Figure 8:**
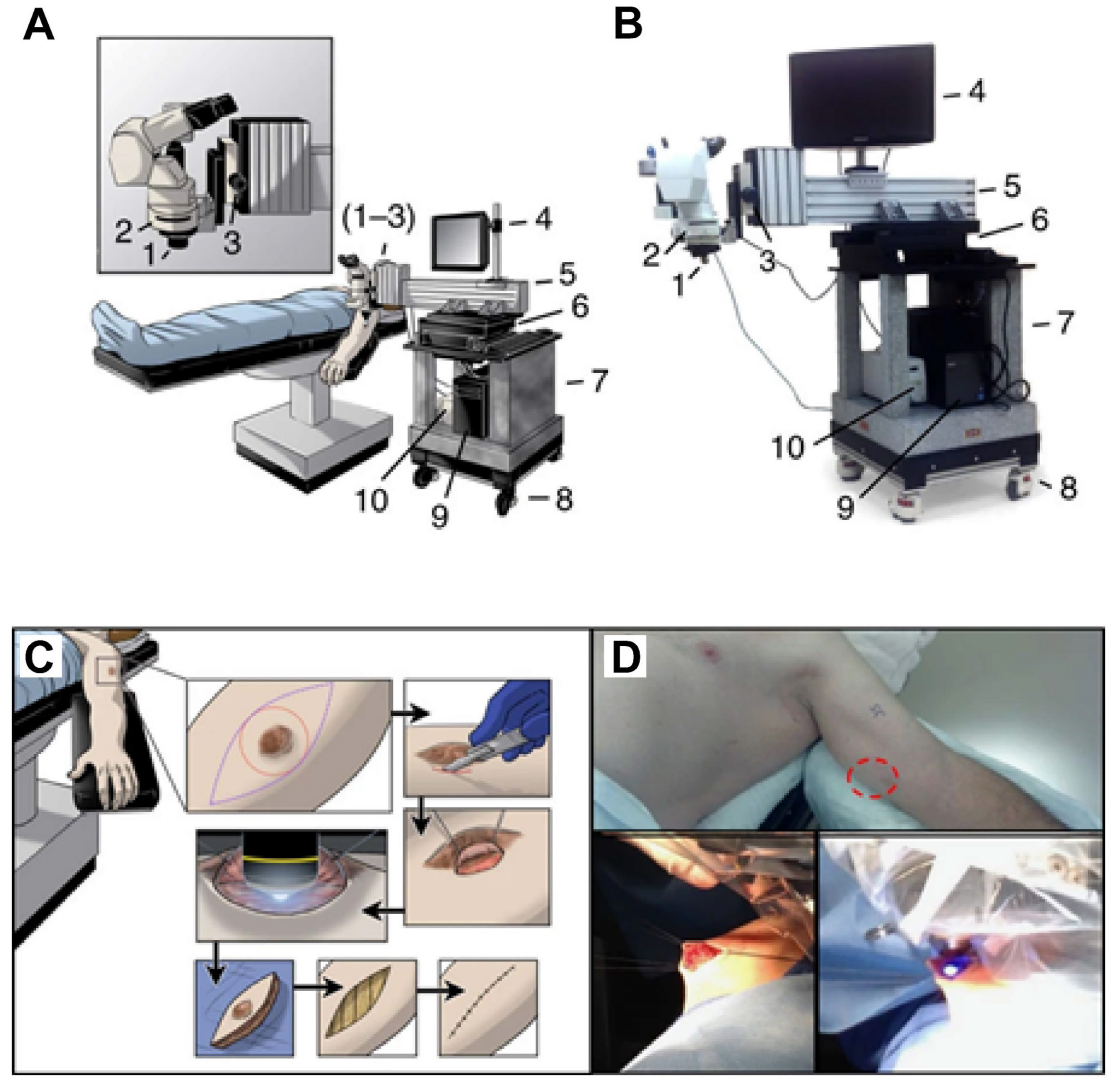
** Clinical IVM. (A).** Schematic and **(B)** photograph of the IVM unit designed as a mobile system for observations in the operating room. Critical systems allowing for stable epifluorescence observation of patient tumors in real-time are highlighted as follows: **(1)** × 10 objective lens (× 100 total including × 10 subjective lens), **(2)** fluorescent filter cube, **(3)** fine focus knob, **(4)** monitor to observe in real time, **(5)** cantilevered arm to extend the system over the patient and hold the apparatus steady, **(6)** heavy movement platform to control fine x-y motion of the system during observation, **(7)** solid granite base to reduce vibrations, **(8)** locking wheels, allowing mobility and stability, **(9)** integrated computer system to record observations and store data and **(10)** fluorescent light source. **(C)** Schematic and **(D)** representative photographs, detailing the surgical procedure to examine tumor microvasculature. Briefly, an incision is made in the skin overlying or adjacent to the tumor (red circle) to allow access for direct tumor observation. The epifluorescent light source is activated and digital recording commences with real time observation of the intravenous injection of fluorescein. Following observation, the access site is closed, and a standard oncologic resection performed. Adapted from 199 with permission, 2016 Springer Nature.

Beyond the preclinical vascular studies, intravital imaging shows promise in visualizing tumor vessels in humans. Intravital imaging has been applied to the endoscopic evaluation of cancers in the gastrointestinal tract (such as in the esophagus 192, 193, stomach 194 and colon 195), the cystoscopic evaluation of bladder urothelial cancer 196, the evaluation of peritoneal carcinomatosis during cytoreductive surgery 197, and, most recently, the evaluation of melanoma 135, 198. An operating room compatible, mobile confocal microscope (Figure 8 A-D) has been used to directly observe patient tumor vasculature associated with primary or in-transit melanomas 199. Consistent with preclinical observations, patient tumor vessels are disorganized and tortuous, and ∼50% of them do not support blood flow. Human tumor vessel diameters are larger than those predicted from immunohistochemistry or preclinical IVM and thereby have lower wall shear stress, influencing drug delivery and cellular immunotherapies. Thus, real-time clinical imaging of living human superficial or endoluminal tumors may be helpful in monitoring local therapies 199.

**Figure 9:**
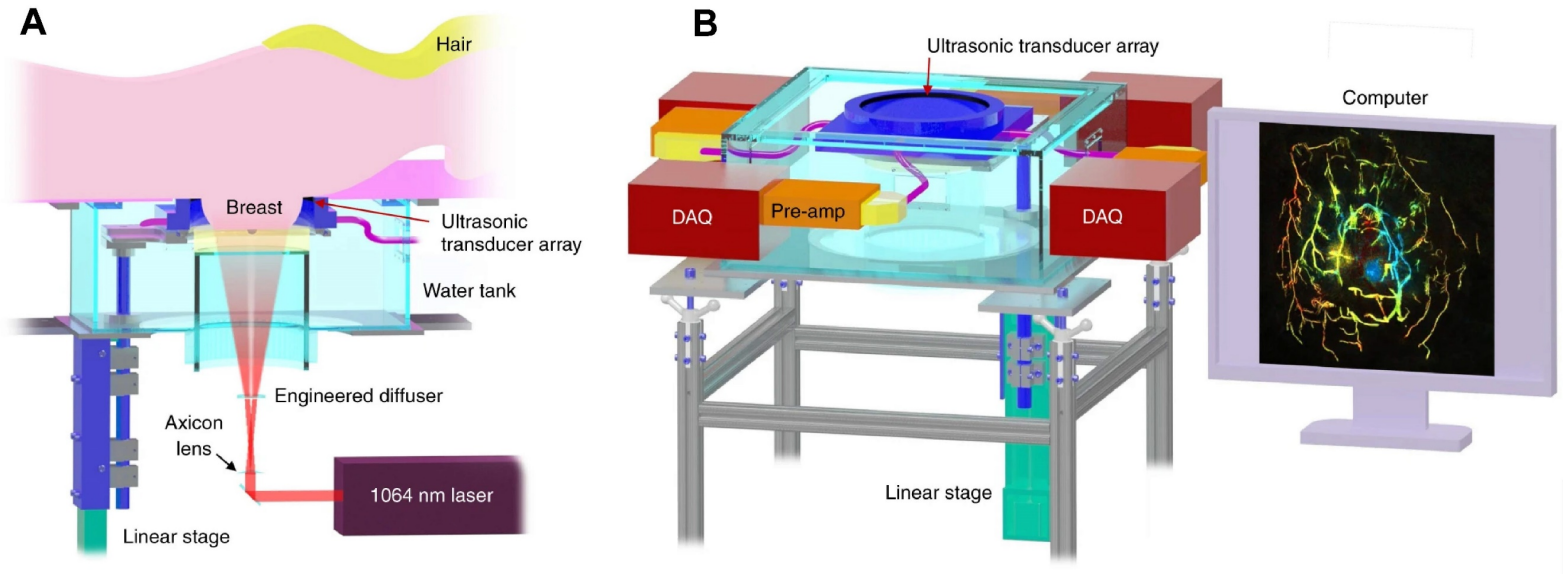
**Clinical PAI.** Representations of the SBH-PACT system. **(A).** Perspective cut-away view of the system with data acquisition components removed. **(B).** Perspective view of the system with patient bed and optical components removed. DAQ: data acquisition system, Pre-amp: pre-amplifier circuits. Adapted from 74 with permission, 2018 Springer Nature.

PAI also has demonstrated great potentials in clinical tumor vascular imaging, especially in breast cancer. PAI with a hemispherical detector array has shown advantages in visualizing fine vasculature, detailed blood-vessel branching structures and morphological characteristics compared over MRI 200. A single-breath-hold photoacoustic computed tomography (SBH-PACT) system developed by Wang LV *et al.* features a deep penetration depth (4 cm) with high spatial and temporal resolutions (255 µm in-plane resolution and a 10 Hz 2D frame rate) (Figure 9) 74. By scanning the entire breast within a single breath hold (~15 s), a volumetric image can be acquired and then is reconstructed utilizing 3D back-projection with negligible breath-induced motion artifacts, visualizing detailed angiographic structures in human breasts. SBH-PACT has demonstrated an early promise in high sensitivity detection of breast cancers in radiographically dense breasts by detecting higher blood vessel densities in tumors than in normal breast tissue 74. In addition, the high imaging speed of SBH-PACT enables dynamic studies, such as photoacoustic elastography, which identifies tumors by their reduced tissue compliance without resorting to ionizing radiation or exogenous contrast 74.

In order to expand clinical applications of intravital imaging, the development of customer-friendly intravital imaging platforms is essential. A portable wi-fi-enabled microscope could conceivably be combined with a smartphone or watch and would be very convenient for monitoring vasculature of superficial cancers reachable by laser light penetration (~2-4 cm), for conditions such as melanoma, oral cancers, and breast cancers. Light weight, multiple-color fluorescence imaging during endoscopy or cystoscopy could visualize blood vessels of gastrointestinal and bladder cancers. Additionally, intravital imaging would allow immediate diagnosis during a surgical operation and help the surgeon precisely excise the cancer tissue. To enable these advances, multi-disciplinary collaborations that bring surgeons and engineers together will be needed. Once sufficient clinical data are accumulated, artificial intelligence-based intravital imaging and analysis tools could be developed, which will help speed the analysis of blood vessels, diagnose, and grade cancers, and predict treatment responses.

In summary, recent advances in intravital imaging technologies, including label-free, multi-modality, and subcellular imaging, and non-invasive methods to visualize tumor vasculatures have a bright future in preclinical and clinical applications. These technical advances promise a diverse toolset with which the examination of tumor vasculature under various conditions, with or without therapies, can be performed. Observations from these studies would form the basis for the next generation of cancer therapies.

## Supplementary Material

Supplementary table.

## Figures and Tables

**Table 1:** Tumor vasculature spatiotemporal measurements.

**Chamber-based animal model**	**Diameter (µm)**	**Intercellular; Intracellular openings (µm)**	**Permeability (×10^7^ cm/sec)**	**Ref**
U87 mouse cranial window		0.07-0.1; ND	3.8±1.2 (2.4-5.0)	18
HCa-I/ST-8 mouse dorsal chamber		0.38-0.55/0.55-0.78; ND	2.06±1.44 (1.60-3.99)/3.73±3.34 (1.67-9.28)	18
LS174T mouse dorsal chamber		0.4-0.6/0.38-0.55; ND	1.24±0.45 (0.56-1.67)	18
MCa-IV mouse dorsal chamber		1.7±0.1; 0.6±0.1	2.5±1.5 (1.2-5.1)	19
P22 rat dorsal chamber	13(6-35)			20
**Xenograft/orthotopic mouse model**	**Diameter (µm)**	**Vessel sprout length (µm)**		**Ref**
RIP-Tag2 pancreatic islet tumors	8±1(5-16)	15±1(3-56)		21
Lewis lung carcinomas	31±2(5-154)	23±4(5-56)		21
MCa-IV mammary Carcinomas	45±6(8-294)	29±4(3-69)		19, 21
Renal clear cell carcinoma, asalioma	6-55	2-52(Intervascular); 34-208(Interbranching)		8
Sarcomas, colon carcinoma	5-80	11-105(Intervascular);11-160(Interbranching)		8

Footnotes: ND: no data

**Table 2:** Tumor blood vessel permeability response to different treatments.

Treatment	Permeability/ extravasation changes	Mechanism (cells, cytokines)	Ref
Angiopoietin-4	↑ 4.5-fold	Activation of TIE2-dependent signaling	149
Sunitinib	No change	EphB4 overexpression and treatment resistance	150
Doxorubicin	↑	MMP9-high myeloid cell recruitment via CCL2/CCR2. Possible VEGF↑ Loss of adherence junctions and decreased pericytes in MMP9 deficient mice	136
Paclitaxel, Doxorubicin/Cyclophosphamide	Transient ↑	TIE2^hi^-TMEM macrophages, MENA↑	138
Nanoparticles (TiO_2_, SiO_2_, Au and Ag)	↑	Disruption of the VE-cadherin-VE-cadherin homophilic interactions at the adherens junctions	140
Nanoparticles (Polymers)	Transient↑	With or without leukocytes nearby	139
Nanoparticles (Au)	↑	Transcellular pathway; Active transcytosis	11
GGT activatable polymer-CPT conjugate nanoparticles	↑	Transcellular pathway: absorption-mediated transcytosis	144
SN38 conjugate polymer nanoparticle	↑	Transcellular pathway: absorption-mediated transcytosis	145
MMP2-responsive polymer-lipid-peptide nanoparticles delivering an antiplatelet antibody R300	↑	Local depletion of tumor-associated platelets that cover vessel wall	142
Inhibition of NO	↓	Restorring normal vasculature	146
MMPs broad-spectrum inhibitor prinomastat	↓		147
Low dose (5-Gy) radiation	Transient↑	TAM, NO signaling and VEGF	151
IFNγ (Overexpressed in tumor cell)	↓	Vascular density decrease (loss of smaller vessels and thinning of larger vessels)	152
TNF (Overexpressed in tumor cell)	↑	Vessels widening and burst	152
FGF-9 (Overexpressed in tumor cell)	n/a	A network of arterioles, bona fide capillaries, and venules; VEGF↓	148, 153
IL-1β (2 μg/ml) IL-1β (1 μg/ml)	↑ -		154
TGF-β inhibitor	↑	Fomation of pericytes in tumor vessels and reduce the solid pressure of tumors	141
PDT with photosensitizer MV6401	↑	Vasoconstriction; thrombus formation	155
Lower dose (0.25 to 0.5 mg/kg) verteporfin or EGFR-targeted nanobody-PDT	↑		156
PDT with verteporfin	↑	Endothelial cell microtubule depolymerization; endothelial intercellular gaps	157
RGD-modified ferritin-based PDT	↑	Larger fenestrae on the endothelial	158
PDT with the lipid- PLGA nanoparticles	↑	Tumor-associated myeloid cells	159
Tumor targeting PIT	↑		160

**Footnote:** FGF-9: fibroblast growth factor 9, EphB4: Ephrin type-B receptor 4, MMP9: matrix metallopeptidase 9, VEGFA: vascular endothelial growth factor A, TMEM: tumor microenvironment of metastasis, MENA: actin-regulatory protein mammalian-enabled, TiO2: titanium dioxide, SiO2: silicon dioxide, Au: gold, Ag: silver, VE-cadherin: vascular endothelial cadherin, GGT: γ-glutamyl transpeptidase, CPT: camptothecin, SN38: 7-ethyl-10-hydroxycamptothecin, NO: nitric oxide, IFNγ: interferon gamma, TNF: tumor necrosis factor, PDT: photodynamic therapy, EGFR: epidermal growth factor receptor, RGD: arginylglycylaspartic acid, PLGA: PEG layer coated poly (lactic-co-glycolic acid), PIT: photoimmunotherapy
