# Supplementary material for: Visualizing vasculature and its response to therapy in the tumor microenvironment
Source: Theranostics. 2023 Sep 25;13(15):5223–46. doi: 10.7150/thno.84947 (PMC10614675; doi:10.7150/thno.84947)
Supplement: Supplementary file 1 — Supplementary table. [file thnov13p5223s1.pdf]

Supporting information for

Visualizing vasculature and its responses to therapies in the tumor microenvironment

Qiaoya Lin[1], Peter L. Choyke[1] & Noriko Sato[1, *]

1. Molecular Imaging Branch, Center for Cancer Research, National Cancer Institutes, National Institutes of Health, Bethesda, Maryland.

***Correspondence:**

Noriko Sato, MD, PhD. Molecular Imaging Branch, Center for Cancer Research, National Cancer Institute, National Institutes of Health, Bethesda, MD 20892

Phone: 1+240-858-3079; Fax: 1+240-541-4526

Email: saton@mail.nih.gov

Supporting Table 1: Current window models for intravital imaging of vasculatures.

Window organ	Surgery (Y/N)	Study period	Tumor model	Blood vessels visualization method	Stabilizer /device	Microscope	Ref
Ear Skin	N	Longitudinal	n/a	FITC-dextran (2000 kDa) and TRITC-ficoll (70 kDa/400 kDa); Dextran (70 kDa/150 kDa/2000 kDa)	n/a	Upright/inverted LSCM and MPLSM	[1, 2]
Lymph node	Y	Short-term	n/a	Dextran (500 or 2,000 kDa)	Superglue fixation; Custom-built microscope stage	Upright MPLSM	[3-6]
s.c./i.m. tumor in flank	Y	Short-term	CT-26-GFP	PE-anti-CD31 (blood vessel) and PE-anti-CD49b (blood flow)	Secured to a microscope slide by sutures/heated microscope stage	Inverted LSCM and MPLSM	[7]
Dorsal skin	Y	Longitudinal	4T1-GFP or BxPC-3-DesRed s.c. tumor (inoculate at DSWC installation or 10 d)	Oxygen-sensing boron NP (hemoglobin saturation) or FITC-Dextran (2MDa)	DSWC with imaging mount	Upright/inverted WFM and LSCM	[8, 9]

Window organ	Surgery (Y/N)	Study period	Tumor model	Blood vessels visualization method	Stabilizer /device	Microscope	Ref
Dorsal skin	Y	Longitudinal	n/a or B16BL6 implanted tumor	FITC-dextran (150 K Da) or eNOStag-GFP (endothelial cells); Cspg4-DsRed (Pericytes)/ Rhodamine PEGylated NPs (blood flow)	Adapted small and light weight-DSWC	WFM or upright MPLSM	[10, 11]
Dorsal skin	Y	Longitudinal	s.c. A-07-GFP or s.c. LLC-DsRed2 tumor	TRITC-dextran (155 KDa) or iron oxide–dextran NPs	MRI-compatible DSWC	MRI and inverted WFM or MPLSM	[12, 13]
Mammary fat pad	Y	Short-term	MMTV-PyMT orthotopic tumor	Rhodamine-dextran (70-kDa) or TRITC-Dextran (155 kDa) and FITC-dextran (10 kDa)	n/a or custom-built rubber pads or circular imaging window frame with cyanoacrylate adhesive	Inverted SDCM and MPLSM	[14-17]
Mammary fat pad	Y	Longitudinal	MTLn3- Dendra2; MTLn3-CFP; MMTV-PyMT-GFP orthotopic tumors	Fluorescent dextran (10Ka)	Coverslip mounted plastic frame (consisting of two plastic rings)	Upright LSCM and MPLSM	[18]
Mammary pad	Y	Longitudinal	MDA-MB-231-GFP; MCF-7-GFP orthotopic tumors	Alexa Fluor 647-BSA	Custom-made MRI-compatible MWC and animal holder	MRI and upright LSCM	[19]

Window organ	Surgery (Y/N)	Study period	Tumor model	Blood vessels visualization method	Stabilizer /device	Microscope	Ref
Brain	Y	Longitudinal	n/a	Texas red dextran, Evans blue, cascade blue dextran, FITC dextran (120 kDa)	Cyanoacrylate glue for fixing the skull and dental cement to cover the exposed skull	LSCM and MPLSM	[20]
Brain	Y	Longitudinal (up to 5 months)	D283-MED-Gluc and D341 s.c. tumors	TRITC dextran (2000 Kda); TRITC–BSA (68,000 MW)	Custom-built stereotactic frame. Cyanoacrylate glue, acrylic powder (1:1) to fix the coverslip-bone	Ultrasonography and MPSLM/OCT	[21]
Brain	Y	Longitudinal	n/a	n/a	Adapted transparent cranial windows with a tunable screw for controlled compression	MRI, MPLSM and CT	[22]
Brain[1,2,3,4]	Y	Longitudinal	n/a	n/a	Thinned skull transcranial window; Reinforced thinned skull window; Transcranial window; Implanted window; Prism-based window; Lens based window	MPLSM/ MPLSM and PET	[23]

Window organ	Surgery (Y/N)	Study period	Tumor model	Blood vessels visualization method	Stabilizer /device	Microscope	Ref
Bone marrow[4]	Y	Longitudinal	Nalm-6 xenograft (DiD or DiR labeled); n/a	Fluorescent cyanine compounds or AlexaFluor750-ICAM-1, VCAM-1, PECAM-1 and P-selectin; Rhodamine B, Texas red and FITC-dextran (70 kDa) and CFDA-SE-RBCs	Skull window; custom-made stereotactic holder	LSCM and MPLSM	[24-26]
Bone marrow	Y	Longitudinal	T-ALL xenograft (PE-human CD45 antibody labeled)	Cy5-Dextran (500 kDa)	Calvarial bone marrow with protective intrasite gel and custom-made stabilizer	Upright LSCM and MPLSM	[27-29]
Bone marrow	Y	Longitudinal	LnCap-mCherry, Du145-mCherry, and PC3-mCherry s.c. tumors	TRITC-BSA (MW 67,000); FITC-labeled dextran (150 Kda); Quantum dots 655	Femur window (filled bilaterally with Ostron Cement-cover slide); Custom-made clamp to fix the chamber	LSCM and MPLSM	[30-33]
Lung	Y	Short-term (Up to 3 h)	n/a	Texas Red dextran (70 Kda), Red, fluorescent microspheres (blood flow)	Thoracic suction window attached to a micro-manipulator on the microscope stage	MPLSM	[34, 35]
Lung	Y	Short-term (Up to 12 h)	EO771-LG-EGFP metastasis	TRITC dextran (155 kD)	Vacuum stabilized window made of Teflon	Inverted MPLSM	[36]

Window organ	Surgery (Y/N)	Study period	Tumor model	Blood vessels visualization method	Stabilizer /device	Microscope	Ref
Lung	Y	Longitudinal (Days to weeks)	E0771–EGFP metastasis	VeCad-tdTomato (endothelia); TRITC dextran (155 kD); 2.5 µm fluorescent microspheres (blood flow)	Implantable lung window	Inverted MPLSM	[37]
Liver	Y	Short-term (up to 4 h)	n/a	Anti-mouse CD31 (PECAM-1) (endothelial cells); Anti-CD41 (platelets).	PBS-soaked Kimwipes to cover the exposed lobe; Custom-made acrylic platform	Inverted LSCM	[38, 39]
Liver	Y	Short-term (Up to 6 h)	SL4-RFP or HT29-RFP metastasis	n/a	Instant adhesive agent and a custom build-organ stabilizing system or agarose embedding	Upright MPLSM	[40, 41]
Liver	Y	Short-term	n/a	n/a	Custom-built imaging box with stabilizer	Inverted LSCM and OR-PAM	[42]
Liver, spleen, kidney, small intestine, Pancreas	Y	Longitudinal	C26–H2B-Dendra2, C26-Dendra2, C26-mCherry, and C26–LifeAct-GFP metastasis	Texas-Red dextran (70 kDa)	Abdominal imaging window with a custom-built imaging box	Inverted MPLSM	[43, 44]

Window organ	Surgery (Y/N)	Study period	Tumor model	Blood vessels visualization method	Stabilizer /device	Microscope	Ref
Pancreas	Y	Longitudinal	PANC-1 orthotopic tumor (4 weeks before window installation)	FITC-dextran (2000 Kda), TRITC-BSA	Abdominal wall window made of titanium circular mount with 8 holes on the edge	IVM	[45]
Pancreas	Y	Longitudinal	Rip1-Tag2 orthotopic tumor; KPC-EGFP orthotopic tumor	VeCad-tdTomato mice; FITC or TRITC-dextran (50 kDa)	Custom-built imaging window with cyanoacrylate, and a custom-made plastic c-clip to stabilize	MPLSM and laser speckles imaging/ MPLSM and MRI	[46, 47]
Ovary	Y	Longitudinal	ES-2-tdTomato or Es-2-eGFP s.c. tumor	FITC-dextran (500 kDa)	Custom-built imaging window with cyanoacrylate, and a custom-made plastic c-clip to stabilize	MPLSM	[48]
Mouse embryo	Y	Longitudinal	n/a	n/a	Custom-built abdominal window	MPLSM	[49]

Footnotes: Mouse models are used except for 1: ferret, 2: non-human primate, 3: pig and 4: rat. CT-26-GFP: mouse colon carcinoma; 4T1-GFP: mouse mammary carcinoma; BxPC-3-DesRed: human pancreatic adenocarcinoma; B16BL6: mouse melanoma; A-07-GFP: human melanoma; LLC-DsRed2: mouse Lewis lung carcinoma; MMTV-PyMT: (mouse mammary tumor virus-polyoma middle tumor-antigen) mouse model of breast cancer; MTLn3-Dentra2/MTLn3-CFP: highly invasive rat breast adenocarcinoma; MDA-MB-231-GFP: human breast adenocarcinoma; MCF-7-GFP: human breast adenocarcinoma; D283-MED-Gluc and D341: human medulloblastoma; Nalm-6: human B cell precursor leukemia; T-All: T-cell acute lymphoblastic leukemia; LnCap-mcherry: (Lymph Node Carcinoma of the Prostate) human prostate adenocarcinoma; Du145-mCherry: human prostate carcinoma with androgen receptor (AR) expression; PC3-mCherry: human prostate adenocarcinoma; EO771-LG-EGFP/EO771-EGFP: mouse mammary carcinoma; SL4-RFP: mouse colon carcinoma; HT29-RFP: human colorectal adenocarcinoma; C26-H2B-Dendra 2/C26-Dendra2/C26-mCherry/C26-LifeAct-GFP: mouse colon carcinoma; PNAC-1: pancreas ductal adenocarcinoma; Rip1-Tag2 mice: mouse pancreatic islet cell carcinoma; KPC-EGFP: mouse pancreatic ductal adenocarcinoma; ES-2-tdTomato or ES-2-eGFP: human ovarian carcinoma.

References

1. Honkura N, Richards M, Laviña B, Sáinz-Jaspeado M, Betsholtz C, Claesson-Welsh L. Intravital imaging-based analysis tools for vessel identification and assessment of concurrent dynamic vascular events. Nat Commun. 2018; 9: 2746.
2. Egawa G, Ono S, Kabashima K. Intravital Imaging of Vascular Permeability by Two-Photon Microscopy. Methods Mol Biol. 2021; 2223: 151-7.
3. Mempel TR, Henrickson SE, von Andrian UH. T-cell priming by dendritic cells in lymph nodes occurs in three distinct phases. Nature. 2004; 427: 154-9.
4. von Andrian UH, Mempel TR. Homing and cellular traffic in lymph nodes. Nat Rev Immunol. 2003; 3: 867-78.
5. Lin Q, Liu Z, Luo M, Zheng H, Qiao S, Han C, et al. Visualizing DC morphology and T cell motility to characterize DC-T cell encounters in mouse lymph nodes under mTOR inhibition. Sci China Life Sci. 2019; 62: 1168-77.
6. Miller MJ, Wei SH, Parker I, Cahalan MD. Two-photon imaging of lymphocyte motility and antigen response in intact lymph node. Science. 2002; 296: 1869-73.
7. Turk M, Naumenko V, Mahoney DJ, Jenne CN. Tracking Cell Recruitment and Behavior within the Tumor Microenvironment Using Advanced Intravital Imaging Approaches. Cells. 2018; 7.
8. Palmer GM, Fontanella AN, Shan S, Hanna G, Zhang G, Fraser CL, et al. In vivo optical molecular imaging and analysis in mice using dorsal window chamber models applied to hypoxia, vasculature and fluorescent reporters. Nat Protoc. 2011; 6: 1355-66.
9. Maeda A, DaCosta RS. Optimization of the dorsal skinfold window chamber model and multi-parametric characterization of tumor-associated vasculature. Intravital. 2014; 3: e27935.
10. Schreiter J, Meyer S, Schmidt C, Schulz RM, Langer S. Dorsal skinfold chamber models in mice. GMS Interdiscip Plast Reconstr Surg DGPW. 2017; 6: Doc10.
11. Seynhaeve ALB, ten Hagen TLM. An adapted dorsal skinfold model used for 4D intravital followed by whole-mount imaging to reveal endothelial cell–pericyte association. Sci Rep. 2021; 11: 20389.
12. Gaustad JV, Brurberg KG, Simonsen TG, Mollatt CS, Rofstad EK. Tumor vascularity assessed by magnetic resonance imaging and intravital microscopy imaging. Neoplasia. 2008; 10: 354-62.
13. Erten A, Wrasidlo W, Scadeng M, Esener S, Hoffman RM, Bouvet M, et al. Magnetic resonance and fluorescence imaging of doxorubicin-loaded nanoparticles using a novel in vivo model. Nanomedicine. 2010; 6: 797-807.
14. Ewald AJ, Werb Z, Egeblad M. Dynamic, long-term in vivo imaging of tumor-stroma interactions in mouse models of breast cancer using spinning-disk confocal microscopy. Cold Spring Harb Protoc. 2011; 2011: pdb.top97.
15. Nakasone ES, Askautrud HA, Egeblad M. Live imaging of drug responses in the tumor microenvironment in mouse models of breast cancer. J Vis Exp. 2013: e50088.
16. Harney AS, Wang Y, Condeelis JS, Entenberg D. Extended Time-lapse Intravital Imaging of Real-time Multicellular Dynamics in the Tumor Microenvironment. J Vis Exp. 2016.
17. Karagiannis GS, Pastoriza JM, Borriello L, Jafari R, Coste A, Condeelis JS, et al. Assessing Tumor Microenvironment of Metastasis Doorway-Mediated Vascular Permeability Associated with Cancer Cell Dissemination using Intravital Imaging and Fixed Tissue Analysis. J Vis Exp. 2019.
18. Kedrin D, Gligorijevic B, Wyckoff J, Verkhusha VV, Condeelis J, Segall JE, et al. Intravital imaging of metastatic behavior through a mammary imaging window. Nat Methods. 2008; 5: 1019-21.
19. Schafer R, Leung HM, Gmitro AF. Multi-modality imaging of a murine mammary window chamber for breast cancer research. Biotechniques. 2014; 57: 45-50.
20. Tong L, Hill RA, Damisah EC, Murray KN, Yuan P, Bordey A, et al. Imaging and optogenetic modulation of vascular mural cells in the live brain. Nat Protoc. 2021; 16: 472-96.
21. Askoxylakis V, Badeaux M, Roberge S, Batista A, Kirkpatrick N, Snuderl M, et al. A cerebellar window for intravital imaging of normal and disease states in mice. Nat Protoc. 2017; 12: 2251-62.

22. Nia HT, Datta M, Seano G, Zhang S, Ho WW, Roberge S, et al. In vivo compression and imaging in mouse brain to measure the effects of solid stress. Nat Protoc. 2020; 15: 2321-40.
23. Cramer SW, Carter RE, Aronson JD, Kodandaramaiah SB, Ebner TJ, Chen CC. Through the looking glass: A review of cranial window technology for optical access to the brain. J Neurosci Methods. 2021; 354: 109100.
24. Sipkins DA, Wei X, Wu JW, Runnels JM, Côté D, Means TK, et al. In vivo imaging of specialized bone marrow endothelial microdomains for tumour engraftment. Nature. 2005; 435: 969-73.
25. Kikuta J, Ishii M. Bone Imaging: Osteoclast and Osteoblast Dynamics. Methods Mol Biol. 2018; 1763: 1-9.
26. Jung Y, Spencer JA, Raphael AP, Wu JW, Alt C, Runnels JR, et al. Intravital Imaging of Mouse Bone Marrow: Hemodynamics and Vascular Permeability. In: Ishii M, editor. Intravital Imaging of Dynamic Bone and Immune Systems : Methods and Protocols. New York, NY: Springer New York; 2018. p. 11-22.
27. Scott MK, Akinduro O, Lo Celso C. In vivo 4-dimensional tracking of hematopoietic stem and progenitor cells in adult mouse calvarial bone marrow. J Vis Exp. 2014: e51683.
28. Hawkins ED, Duarte D, Akinduro O, Khorshed RA, Passaro D, Nowicka M, et al. T-cell acute leukaemia exhibits dynamic interactions with bone marrow microenvironments. Nature. 2016; 538: 518-22.
29. Duarte D, Hawkins ED, Akinduro O, Ang H, De Filippo K, Kong IY, et al. Inhibition of Endosteal Vascular Niche Remodeling Rescues Hematopoietic Stem Cell Loss in AML. Cell Stem Cell. 2018; 22: 64-77.e6.
30. Hansen-Algenstaedt N, Schaefer C, Wolfram L, Joscheck C, Schroeder M, Algenstaedt P, et al. Femur window--a new approach to microcirculation of living bone in situ. J Orthop Res. 2005; 23: 1073-82.
31. Mussawy H, Viezens L, Schroeder M, Hettenhausen S, Sündermann J, Wellbrock J, et al. The bone microenvironment promotes tumor growth and tissue perfusion compared with striated muscle in a preclinical model of prostate cancer in vivo. BMC Cancer. 2018; 18: 979.
32. Chen Y, Maeda A, Bu J, DaCosta R. Femur Window Chamber Model for In Vivo Cell Tracking in the Murine Bone Marrow. J Vis Exp. 2016.
33. Reismann D, Stefanowski J, Günther R, Rakhymzhan A, Matthys R, Nützi R, et al. Longitudinal intravital imaging of the femoral bone marrow reveals plasticity within marrow vasculature. Nat Commun. 2017; 8: 2153.
34. Looney MR, Thornton EE, Sen D, Lamm WJ, Glenny RW, Krummel MF. Stabilized imaging of immune surveillance in the mouse lung. Nat Methods. 2011; 8: 91-6.
35. Lamm WJ, Bernard SL, Wagner WW, Jr., Glenny RW. Intravital microscopic observations of 15-microm microspheres lodging in the pulmonary microcirculation. J Appl Physiol (1985). 2005; 98: 2242-8.
36. Entenberg D, Rodriguez-Tirado C, Kato Y, Kitamura T, Pollard JW, Condeelis J. In vivo subcellular resolution optical imaging in the lung reveals early metastatic proliferation and motility. Intravital. 2015; 4: 1-11.
37. Entenberg D, Voiculescu S, Guo P, Borriello L, Wang Y, Karagiannis GS, et al. A permanent window for the murine lung enables high-resolution imaging of cancer metastasis. Nat Methods. 2018; 15: 73-80.
38. Marques PE, Antunes MM, David BA, Pereira RV, Teixeira MM, Menezes GB. Imaging liver biology in vivo using conventional confocal microscopy. Nat Protoc. 2015; 10: 258-68.
39. Babes L, Kubes P. Visualizing the Tumor Microenvironment of Liver Metastasis by Spinning Disk Confocal Microscopy. Methods Mol Biol. 2016; 1458: 203-15.
40. Tanaka K, Morimoto Y, Toiyama Y, Okugawa Y, Inoue Y, Uchida K, et al. Intravital dual-colored visualization of colorectal liver metastasis in living mice using two photon laser scanning microscopy. Microsc Res Tech. 2012; 75: 307-15.

41. Heymann F, Niemietz PM, Peusquens J, Ergen C, Kohlhepp M, Mossanen JC, et al. Long term intravital multiphoton microscopy imaging of immune cells in healthy and diseased liver using CXCR6.Gfp reporter mice. J Vis Exp. 2015.
42. Lin Q, Deng D, Song X, Dai B, Yang X, Luo Q, et al. Self-Assembled "Off/On" Nanopomegranate for In Vivo Photoacoustic and Fluorescence Imaging: Strategic Arrangement of Kupffer Cells in Mouse Hepatic Lobules. ACS Nano. 2019; 13: 1526-37.
43. Ritsma L, Steller EJ, Beerling E, Loomans CJ, Zomer A, Gerlach C, et al. Intravital microscopy through an abdominal imaging window reveals a pre-micrometastasis stage during liver metastasis. Sci Transl Med. 2012; 4: 158ra45.
44. Ritsma L, Steller EJ, Ellenbroek SI, Kranenburg O, Borel Rinkes IH, van Rheenen J. Surgical implantation of an abdominal imaging window for intravital microscopy. Nat Protoc. 2013; 8: 583-94.
45. Tsuzuki Y, Carreira CM, Bockhorn M, Xu L, Jain RK, Fukumura D. Pancreas Microenvironment Promotes VEGF Expression and Tumor Growth: Novel Window Models for Pancreatic Tumor Angiogenesis and Microcirculation. Lab Invest. 2001; 81: 1439-51.
46. Martins AF, Clavijo Jordan V, Bochner F, Chirayil S, Paranawithana N, Zhang S, et al. Imaging Insulin Secretion from Mouse Pancreas by MRI Is Improved by Use of a Zinc-Responsive MRI Sensor with Lower Affinity for Zn(2+) Ions. J Am Chem Soc. 2018; 140: 17456-64.
47. Bochner F, Mohan V, Zinger A, Golani O, Schroeder A, Sagi I, et al. Intravital imaging of vascular anomalies and extracellular matrix remodeling in orthotopic pancreatic tumors. Int J Cancer. 2020; 146: 2209-17.
48. Bochner F, Fellus-Alyagor L, Kalchenko V, Shinar S, Neeman M. A Novel Intravital Imaging Window for Longitudinal Microscopy of the Mouse Ovary. Sci Rep. 2015; 5: 12446.
49. Huang Q, Cohen MA, Alsina FC, Devlin G, Garrett A, McKey J, et al. Intravital imaging of mouse embryos. Science. 2020; 368: 181-6.
